# Methane-cycling microbial communities from Amazon floodplains and upland forests respond differently to simulated climate change scenarios

**DOI:** 10.1186/s40793-024-00596-z

**Published:** 2024-07-17

**Authors:** Júlia B. Gontijo, Fabiana S. Paula, Wanderlei Bieluczyk, Aline G. França, Deisi Navroski, Jéssica A. Mandro, Andressa M. Venturini, Fernanda O. Asselta, Lucas W. Mendes, José M. S. Moura, Marcelo Z. Moreira, Klaus Nüsslein, Brendan J. M. Bohannan, Paul L. E. Bodelier, Jorge L. Mazza Rodrigues, Siu M. Tsai

**Affiliations:** 1 Centro de Energia Nuclear na Agricultura, Universidade de São Paulo, Piracicaba, SP Brazil; 2 Department of Land, Air and Water Resources, University of California, Davis, CA USA; 3 Department of Biology, Stanford University, Stanford, CA USA; 4 Instituto de Formação Interdisciplinar e Intercultural, Universidade Federal do Oeste do Pará, Santarém, PA Brazil; 5 Department of Microbiology, University of Massachusetts, Amherst, MA USA; 6 Department of Biology, Institute of Ecology and Evolution, University of Oregon, Eugene, OR USA; 7 Netherlands Institute of Ecology, NIOO-KNAW, Wageningen, GE The Netherlands; 8 Environmental Genomics and Systems Biology Division, Lawrence Berkeley National Laboratory, Berkeley, CA USA

**Keywords:** Amazon rainforest, Wetlands, Global warming, 16S rRNA sequencing, qPCR, Methanogens, Methanotrophs

## Abstract

Seasonal floodplains in the Amazon basin are important sources of methane (CH_4_), while upland forests are known for their sink capacity. Climate change effects, including shifts in rainfall patterns and rising temperatures, may alter the functionality of soil microbial communities, leading to uncertain changes in CH_4_ cycling dynamics. To investigate the microbial feedback under climate change scenarios, we performed a microcosm experiment using soils from two floodplains (i.e., Amazonas and Tapajós rivers) and one upland forest. We employed a two-factorial experimental design comprising flooding (with non-flooded control) and temperature (at 27 °C and 30 °C, representing a 3 °C increase) as variables. We assessed prokaryotic community dynamics over 30 days using 16S rRNA gene sequencing and qPCR. These data were integrated with chemical properties, CH_4_ fluxes, and isotopic values and signatures. In the floodplains, temperature changes did not significantly affect the overall microbial composition and CH_4_ fluxes. CH_4_ emissions and uptake in response to flooding and non-flooding conditions, respectively, were observed in the floodplain soils. By contrast, in the upland forest, the higher temperature caused a sink-to-source shift under flooding conditions and reduced CH_4_ sink capability under dry conditions. The upland soil microbial communities also changed in response to increased temperature, with a higher percentage of specialist microbes observed. Floodplains showed higher total and relative abundances of methanogenic and methanotrophic microbes compared to forest soils. Isotopic data from some flooded samples from the Amazonas river floodplain indicated CH_4_ oxidation metabolism. This floodplain also showed a high relative abundance of aerobic and anaerobic CH_4_ oxidizing Bacteria and Archaea. Taken together, our data indicate that CH_4_ cycle dynamics and microbial communities in Amazonian floodplain and upland forest soils may respond differently to climate change effects. We also highlight the potential role of CH_4_ oxidation pathways in mitigating CH_4_ emissions in Amazonian floodplains.

**Supplementary Information:**

The online version contains supplementary material available at 10.1186/s40793-024-00596-z.

## Introduction

The Amazon floodplains are lowland ecosystems that experience large seasonal variations in rainfall, leading to periodic flooding events [89]. The Amazon basin receives high annual precipitation, which is irregularly distributed between rainy and dry seasons, and fluctuations in river flow result in flooding of an area of more than 800,000 km^2^ during at least six months (depending on the region) every year [31, 36]. These areas represent 20% of the Amazon rainforest and play an essential role in global biogeochemistry, making significant contributions to the carbon (C) cycle and methane (CH_4_) budget [4, 23, 67]. Recent models suggest that Amazonian floodplains contribute up to 29% of the total global wetland CH_4_ emissions [95]. Nearby upland Amazon forests, on the other hand, act as potential atmospheric sinks for CH_4_ [57, 82].

Globally, atmospheric CH_4_ concentration has increased by approximately 18% over the last four decades, posing an environmental threat due to its warming potential, which is 27-fold greater than that of CO_2_ over 100 years [34, 62]. Consequently, the escalating emissions of CH_4_ have made a significant contribution to alterations in the planet’s climate, accounting for 30 to 50% of the increase in global temperatures [34]. Projections for this century also suggest that the Amazon basin may experience an increase in atmospheric temperature ranging from 1.8 to 5.1 °C, with an average of 3.3 °C [48], in addition to shifts in rainfall patterns, resulting in more extreme wet and dry seasons [19, 77, 81].

The emission of CH_4_ is the result of both production and oxidation processes [49]. When oxygen is absent, methane (CH_4_) is produced as the final step in the microbial breakdown of complex organic matter, that involves methanogenic archaea utilizing substrates such as H_2_, CO_2_, acetate, and methylated compounds. Regardless of the specific pathway, all methanogens possess the same terminal enzyme methyl-coenzyme M reductase (MCR), which can be detected through the presence of the *mcr*A gene [46]. In contrast, CH_4_ is oxidized by aerobic and anaerobic methanotrophs, which utilize CH_4_ as an energy and major carbon source, playing a crucial role in reducing CH_4_ emissions. Furthermore, some methanotrophs have the capability to utilize multi-carbon sources in addition to CH_4_ [41]. Aerobic methanotrophic bacteria, categorized as Type I, Type II and Type III, are found in both terrestrial and aquatic environments [26, 75]. The key enzyme responsible for bacterial CH_4_ oxidation is methane monooxygenase (MMO), which exists in both particulate membrane-bound (pMMO) and soluble (sMMO) forms [14]. The gene *pmo*A is commonly used to identify aerobic methanotrophs in environmental samples [40]. Anaerobic CH_4_ oxidation typically occurs under oxygen-deprived conditions, with electron acceptors other than oxygen, performed by some Archaea and Bacteria. This process can occur through reversed methanogenesis or coupled with the reduction of metals such as Fe(III) and Mn(IV) in Archaea and by nitrite-dependent oxidation in Bacteria [17, 96].

Atmospheric temperature and seasonal flooding conditions are known to influence the composition of CH_4_-cycling microbial communities in soil. However, the nature and extent of these influences may be driven by an intricate interplay of environmental factors [30, 69, 72]. Several studies have investigated the role of CH_4_-related microbial communities in Amazonian floodplains and upland soils [2, 5, 25, 57, 82]. For instance, Venturini et al. [82] found that an increase in soil moisture led to changes in the CH_4_-related microbial communities and increased CH_4_ emissions in Amazonian soils. However, none of these studies manipulated temperature and flooding to simulate climate change scenarios in order to understand the effects of these factors, both individually or in combination.

Our aim was to investigate how predicted climate changes in the Amazon basin, specifically the combination of increased temperatures with either flooded or dry conditions, impact the composition of soil microbial communities and, consequently, the net flux of CH_4_ from floodplain and upland forest soils. We, therefore, performed a 30-day microcosm experiment to study the effects of temperature rise (simulating a warmer scenario) and flooding (simulating the wet and dry seasons) on the presence and abundance of CH_4_ producing and consuming microbes related to net surface CH_4_ fluxes. We used 16S rRNA high-throughput sequencing and real-time quantitative PCR to identify and quantify methanogens (CH_4_ producers) and aerobic/anaerobic methanotrophs (CH_4_ consumers). We analyzed environmental parameters, such as soil chemical characterization, CH_4_ fluxes and isotopic signatures of the CH_4_ source (δ^13^C-CH_4_ and δ^2^H-CH_4_), to determine the relationship between CH_4_-associated biogeochemical processes and changes in the microbial community composition and abundance across our experimental treatments. Our results represent a step forward in understanding how the modulation of soil CH_4_-cycling microbial communities and in combination with environmental factors in Amazon soils, impact net CH_4_ fluxes in response to climate change in the Amazon Basin.

## Methods

### Site description, soil sampling, and chemical and physical analysis

The studied sites are located in the Central-West region of the State of Pará, in the municipalities of Santarem and Belterra, Brazil (Supplementary Figure S1). The regional climate is classified as Am (Köppen), tropical humid, with a mean annual temperature of 26 ± 2 °C, rainfall regime with a dry season (from July to November), and total precipitation above 2500 mm per year [1].

Soil sampling was carried out in November 2018, during what is typically considered the dry season, characterized by the absence of a water column in the floodplain sites. Site selection took into consideration the contrasting chemical properties of the soils in the two floodplains, which are influenced by different rivers. One site was located at the intersection of the Amazonas and Tapajós rivers (FP1, 2°22′44.8″ S 54°44′21.1″ W), while the other was located along the Tapajós river (FP2, 2°49′04.6″ S 55°02′04.6″ W). Additionally, soil sampling was also performed in an upland forest site (PFO, 2°51′19.6″ S 54°57′30.1″ W).

In each site, soil samples were collected from a transect consisting of four equally spaced points (20 m between the sampling points in the floodplains and 50 m in the upland forest). At each point, the litter layer was removed, and approximately 5 kg of 0–10 cm deep soil samples were collected. The collected material was transported to the Cellular and Molecular Biology Laboratory of the Center for Nuclear Energy in Agriculture, University of São Paulo (CENA/USP) and stored at 16 °C in the dark until the experiment was carried out, within two weeks. After the storage period, the samples were homogenized and sieved through an 8 mm mesh sieve to remove litter material in preparation for the experiment assembly.

A subset of the soil samples (300 g) was sent to the Pirasolo Laboratório Agrotécnico Piracicaba Ltda (Piracicaba, Brazil) for chemical and physical analysis according to the methods described in Camargo et al. [7]: pH in water (H_2_O); available phosphorus (P), and exchangeable potassium (K), calcium (Ca), and magnesium (Mg), by extraction in ion exchange resin; sulfate (SO_4_^2−^) by turbidimetry and extraction with calcium phosphate 0.01 mol L^−1^; free aluminum (Al) by extracting 1 mol L^−1^ potassium chloride; organic matter (OM) by the dichromate/titrimetric method; total nitrogen (N) by sulfuric/Kjeldahl digestion method; boron (B) by hot water extraction; copper (Cu), iron (Fe), manganese (Mn) and zinc (Zn) extracted by diethylenetriaminepentaacetic acid-triethanolamine (DTPA-TEA) extractor (pH 7.3). Physical analysis of the samples was also carried out for determination of the sand content by weighing and silt and clay by the use of the densimeter.

### Microcosm experimental design

The microcosm experiment had a 3 × 2 × 2 factorial design, whereby soil samples from the three sites (two floodplains and one upland forest) were subjected to two temperatures (27 and 30 °C) and two flooding conditions (wet and dry). Each treatment had four replicates, representing the original field sampling points. Each treatment was established in UV-sterilized 1.5 L glass jars (10.5 × 10.5 × 20 cm) filled with 400 g of fresh soil each. The control temperature of 27 °C was determined since it was the average air temperature measured during the sampling campaign, and the increase of 3 °C aligns with the predicted average temperature rise according to Malhi et al. [48]. Similarly, the moisture level of the dry treatments was set at 30% by weight, as it reflected the average soil moisture in PFO in the field. The water column for the wet treatments were obtained by adding 300 mL of sterile ultrapure water type 1 (Milli-Q®, Merck & Co. Inc., Rahway, NJ, USA). Before starting the 30-day incubation, the samples were kept at 30% moisture and 27 °C for 7 days for soil acclimatization.

The experiment was carried out in two identical Biochemical Oxygen Demand (B.O.D.) incubators for 30 days, one for each temperature. The jars were sealed with moisture-resistant thermoplastic (Parafilm® M, Bemis Company, Inc., WI, EUA) during the incubation to maintain constant moisture and to allow gas exchange throughout the experiment. The soil moisture in each microcosm was monitored during the experiment by weighing and, when needed, corrected with sterile ultrapure water type 1 (Milli-Q®, Merck & Co. Inc., Rahway, NJ, USA) using a sterile spray bottle, followed by homogenization.

The jar lids were previously altered to allow gas sampling after their closing for the CH_4_ measurements (Supplementary Figure S2). CH_4_ and soil samples were collected from each microcosm at time zero (0), followed by changing the treatment conditions in the corresponding jars and then sampling on days 1, 3, 6, 9, 13, 17, 23, and 30. The CH_4_ measurements were performed for 10 min after removing the moisture-resistant thermoplastic (Parafilm® M, Bemis Company, Inc., WI, EUA) and closing the jars with the modified lids. Then, the jars were opened for soil sampling, which involved collecting approximately 6 g of soil from each microcosm. The samples were immediately frozen in liquid N_2_ and subsequently stored at − 80 °C. After soil sampling, the microcosms were checked for moisture content and corrected as necessary. Finally, the jars were sealed with moisture-resistant thermoplastic (Parafilm® M, Bemis Company, Inc., WI, USA) until the next sampling day.

### Measurement of CH_4_ fluxes and isotopic signatures

Continuous CH_4_ concentration (ppm) measurements were conducted for 10 min in closed jars using an Ultra-Portable Greenhouse Gas Analyzer (Los Gatos Research, USA) (Supplementary Fig. 2) with a 10-s interval between measurements, resulting in approximately 60 measurements per jar (600 s in total). During incubation, the analyzer's vacuum pump system sustained a closed-loop flow via two 6-mm tubes, moving air from the glass jar to its laser spectrometer cell. To accurately calculate CH_4_ flux, the concentrations from the first 10 sampling points (100 s) were excluded to reduce fluctuations in the jars’ headspace fluxes (Supplementary Figure S3). Gas fluxes from each jar were calculated based on the concentration in ppm as a function of the incubation time, considering the jar volume, the amount of dry soil in each jar, atmospheric pressure, and air temperature, following:

1 $${\text{ CH}}_{{4\upmu {\text{g}}}} = {\text{CH}}_{{{\text{4ppm}}}} *{\text{CH}}_{{{\text{4MM}}}} *\left[ {\left( {{\text{P}}_{{{\text{atm}}}} {\text{*V}}_{{\text{L}}} } \right)/\left( {{\text{R}}*{\text{T}}_{{\text{K}}} } \right)} \right]$$

2 $${\text{Flux}}\,\upmu {\text{g\,CH}}_{{4}} {\text{g}}_{{{\text{dw}}}}^{ - 1} \,{\text{soil\,h}}^{ - 1} = \left( {{\text{d}}\left[ {{\text{CH}}_{{4\upmu {\text{g}}}} } \right]/{\text{dt}}} \right)*{\text{soil}}_{{{\text{dw}}}} *{60}_{{{\text{min}}}}$$

wherein CH_4µg_ is the CH_4_ mass (µg); CH_4ppm_ is the CH_4_ concentration determined by spectrometry (ppm); CH_4MM_ is the CH_4_ molar mass; P_atm_ is the room pressure (atm); V_L_ is the jar volume (L); R is the ideal gas constant (0.082); T_K_ is the temperature (K); soil_dw_ is the dry soil mass (g_dw_); (d[CH_4µg_]/dt) is the change of CH_4_ µg concentration as a function of time.

Calculations were based on linear regression after a stabilization period of approximately 60 s, excluding the first 100 s to ensure accuracy. Consequently, high R^2^ linear regressions (e.g., greater than 0.9) were obtained (Supplementary Figure S3). The calculation was performed using the first derivative of concentrations and time, with 50 gas concentration readings taken between 100 and 600 s of incubation. For each treatment, daily CH_4_ fluxes were determined by the average of the four replicates per treatment, and total cumulative emissions were determined through the linear interpolation of the daily fluxes between two successive samplings and the sum of the results obtained throughout the experimental period (30 days) [82].

At the end of the 30-day experiment, gas sampling was also performed to analyze the isotopic values of δ^13^C and δ^2^H. Gas samples were collected from the Ultra-Portable Greenhouse Gas Analyzer (Los Gatos Research, USA) outlet connection using a 20 mL syringe and stored in 12 mL evacuated glass vials. Isotopes were analyzed at the University of California Davis Stable Isotope Facility, Davis (USA). Stable isotope ratios of carbon (δ^13^C) and deuterium (δ^2^H) in CH_4_ were measured using a Thermo Scientific Precon concentration unit interfaced with a ThermoScientific Delta V Plus isotope ratio mass spectrometer (ThermoScientific, Bremen, Germany), according to standardized procedures [93]. Standard δ notation was used for quantifying CH_4_ isotopic compositions, as the ratio R of ^13^C to ^12^C and ^2^H to ^1^H in the measured sample was expressed as parts per thousand (‰) as a relative difference (δ^13^C or δ^2^H) from the Vienna PeeDee Belemnite (VPDB) international standard material [52], following:

3 $${\text{C:}}\updelta ^{13} {\text{C}}\,(\permil) = \left( {{\text{R\,sample}}/{\text{R\,VPDB}} - 1} \right) \times 1000$$

4 $${\text{H:}}\updelta ^{2} {\text{H}}\,\left( \permil \right) = \left( {{\text{R\,sample}}/{\text{R\,VSMOW}} - 1} \right) \times 1000$$

The isotopic ratios of the δ^13^C and δ^2^H was corrected using the Keeling plot intercept derived technique [38, 52], which uses the correlation of the isotopic composition of the jars and atmospheric CH_4_ (from atmospheric air samples collect at the end of the experiment) and its inverse concentration. For this, the isotopic intercept of the regression line (δ^13^C vs. 1/CH_4_ and δ^2^H vs. 1/CH_4_) was used to derive the isotopic composition of the mean source inside of each jar. The isotopic signatures (the range of δ^13^C and δ^2^H for the CH_4_ production pathways) were estimated according to Chanton et al. [10] and Whiticar [87] (Supplementary Figure S4).

### DNA extraction and sequencing

DNA was extracted from 0.25 g of the soil samples collected on days 0 and 30 of the microcosm experiment using the PowerLyzer PowerSoil DNA Isolation Kit (Qiagen, Hilden, Germany), following the optimized protocol for tropical soils described by Venturini et al. [84]. DNA quantity and quality were assessed in 1% agarose gel and using a Nanodrop 2000c spectrophotometer (Thermo Fisher Scientific Inc., MA, USA) set for determining absorbance at the 230, 260, 280, and 320 nm wavelengths. Purified DNA samples were stored at − 20 °C until processed.

The archaeal and bacterial communities were assessed by high throughput sequencing of the V4 region of the 16S rRNA gene, amplified with the set of primers 515F [68] and 806R [3] (Table 1). Paired-end sequencing, with 2 × 250 bp reads, was performed in an Illumina Miseq platform at NGS Soluções Genômicas (Piracicaba, Brazil), following the standard procedures of the sequencing facility.

**Table 1:** Set of primers and references for each gene used in this study

Gene	Objective	Target group	Primers	References
16S rRNA	Amplicon Sequencing	Bacteria and Archaea	515F	Parada et al. [68]
			806R	Apprill et al. [3]
16S rRNA	qPCR	Total archaeal community	519F	Klindworth et al. [39]
			915R	Stahl and Amman [78]
16S rRNA	qPCR	Total bacterial community	515F	Caporaso et al. [8]
			806R	
*mcr*A	qPCR	Methanogenic Archaea	mlas-mod-F	Steinberg and Regan [79]
			mcrA-rev	
*pmo*A	qPCR	Methanotrophic Bacteria	A189f	Holmes et al. [33]
			MB661r	Costello and Lidstrom [11]

### Abundance of prokaryotes and methane-cycling microbes

Quantitative PCR (qPCR) was used to quantify the archaeal and bacterial 16S rRNA genes, as well as the genes associated with CH_4_ cycling: *mcr*A which encodes a subunit of methyl coenzyme-M reductase in methanogens and *pmo*A which encodes a subunit of particulate methane monooxygenase in methanotrophs (Table 1). For each gene, a standard curve was established from 10^0^ to 10^6^ copies of the gene. Target genes were previously obtained by PCR from the genomic DNA of *Methanolinea mesofila* (DSMZ 23604) for the Archaeal 16S rRNA and *mcr*A and *Methylosinus sporium* (DSMZ 17706) for the Bacterial 16S rRNA and *pmo*A. The product size of each target gene was checked on 1% agarose gel. The qPCR was performed in triplicate for each sample on a StepOne Plus cycler (Thermo Fisher Scientific, Waltham, MA, USA), with a final volume of 10 μL, containing 5 μL of SYBR Green ROX qPCR (Thermo Fisher Scientific Inc., MA, USA), 1 μL of each primer (5 pmol), 1 μL of soil DNA (adjusted to 10 ng/μL), 0.2 μL of bovine serum albumin (20 mg/mL) (Sigma-Aldrich, San Luis, MO, USA), and 1.8 μL of sterile ultrapure water.

### Bioinformatics and statistical analyses

The bioinformatics and statistical analyses were performed on R 4.0.5 [73]. Raw sequences were processed to infer amplicon sequence variants (ASVs) with the DADA2 1.9.3 package [6]. Approximately 7 million reads were obtained. Forward and reverse reads with a phred score > 20 were truncated at positions 230 and 220 bp, respectively. Sequences were error-corrected, dereplicated, merged, and chimera-filtered. After quality control, 5.5 million sequences with an average length of 269 bp were obtained. The ASV counts were rarefied to 29,300 sequences per sample using phyloseq 1.34.0 [53]. Taxonomy was assigned using the SILVA database (release 138.1, 27.08.2020), and the ASVs classified the same taxonomic groups were summarized at genus level.

Statistical analyses and graphical visualization were carried out using vegan 2.5–1 [64], ARTool 0.10.5 [37], lsmeans 2.30–0 [42], dunn.test 1.3.5 [15], and ggplot2 3.1.0 [88] packages. Shapiro–Wilk normality test and Levene's homogeneity test were performed to define the most appropriate statistical test to be used to detect significant differences among treatments. Kruskal–Wallis with post-hoc Dunn’s test was used to determine statistical differences among the soil chemical properties of the studied areas. Nonmetric multidimensional scaling (NMDS) and permutational multivariate analysis of variance (PERMANOVA) were used to assess the similarities among samples regarding soil chemical properties (Gower distance) and soil microbial community composition (Bray–Curtis distance). In addition, envfit analysis was performed to fit environmental vectors onto an ordination, identifying the chemical properties correlated with each site.

The niche occupancy, i.e., the percentage of generalists and specialists in the different treatments per site, was verified by the multinomial species classification method (CLAM) (default parameters, individual test (alpha) of 0.05, and a coverage limit of 10). Microbial taxa with reported methanogenic or methanotrophic capabilities were manually filtered based on the presence of *mcr*ABC genes for methanogens and *pmo*CAB and/or *mmo*X for methanotrophs available for search in ANOTREE [54]. Two-way ANOVA of aligned rank transformed data was used to investigate the effect of the treatments (temperature × flooding) on each studied site’s soil on the cumulative CH_4_ fluxes. Three-way ANOVA of aligned rank transformed data was performed on the qPCR results, and the relative abundance of methanogens and methanotrophs (sampling day × temperature × flooding).

## Results

### Chemical and physical characterization of the studied sites

**Fig. 1:**
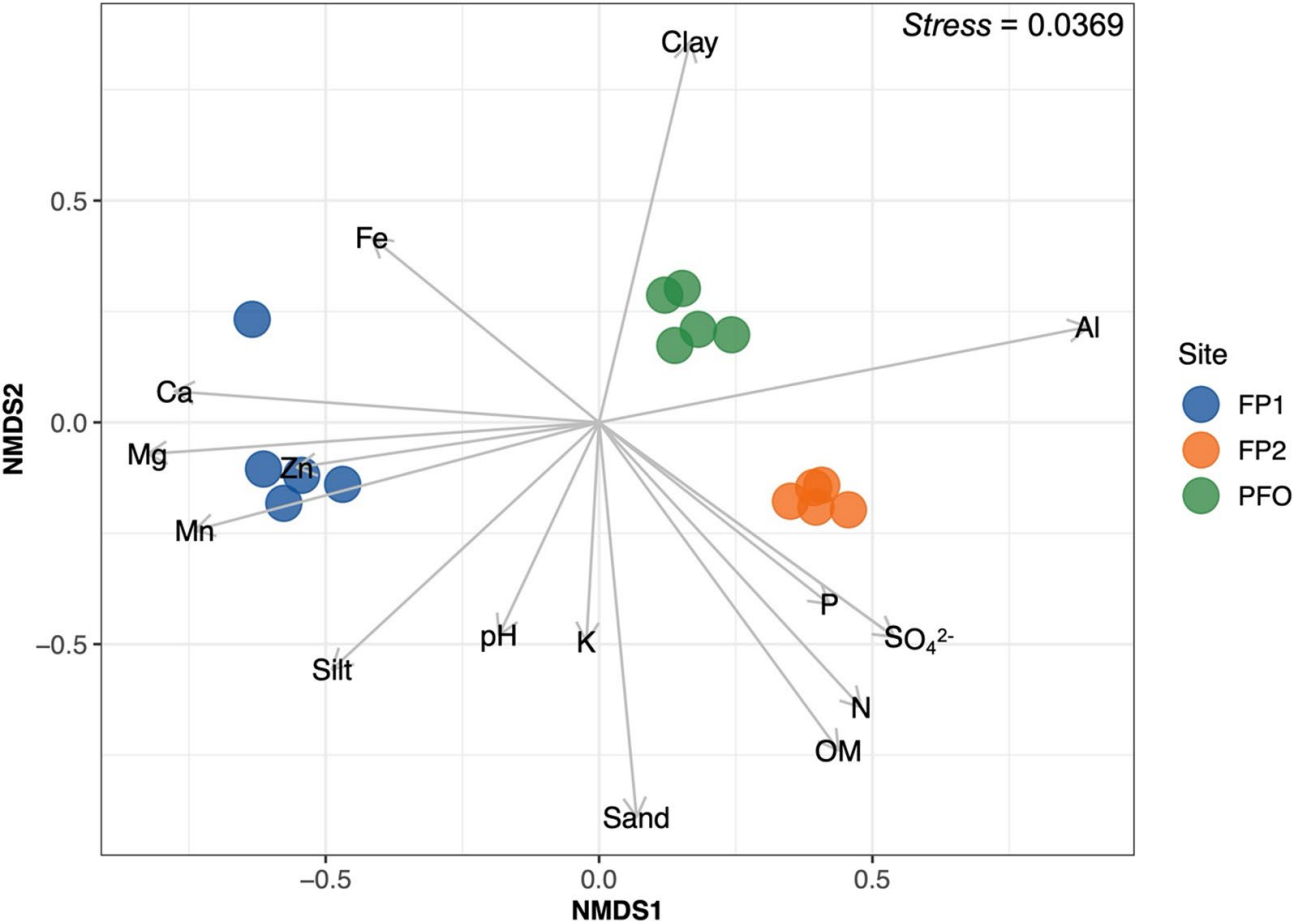
Clustering of the chemical and physical properties of the floodplain (FP1 and FP2) and upland forest (PFO) soils used for the microcosm experiment. Plot is based on the non-metric multidimensional scaling (NMDS) using the Gower distance index. Only environmental factors with significant correlation (p < 0.05) are displayed as vectors

NMDS ordination and envfit analysis were used to verify how the samples from the studied sites clustered based on the chemical and physical characterization of the soils, followed by PERMANOVA similarity analysis. The study areas presented distinct belowground chemical and physical patterns (R^2^ = 0.918, p < 0.001), forming well-defined clusters (Fig. 1). Soils from FP1 were related to higher levels of Fe, Ca, Mg, Mn, Zn, and silt, while FP2 had higher amounts of P, N, SO_4_^2−^, OM, and sand. The upland forest soil was characterized as very clayey (clay contents ranging from 72 to 88%) and with low pH values (< 4.1). Mean, standard deviations, and further statistical results of chemical and physical soil parameters are available in Supplementary Table S2.

### Daily and cumulative CH_4_ fluxes and stable isotopes analysis

**Fig. 2:**
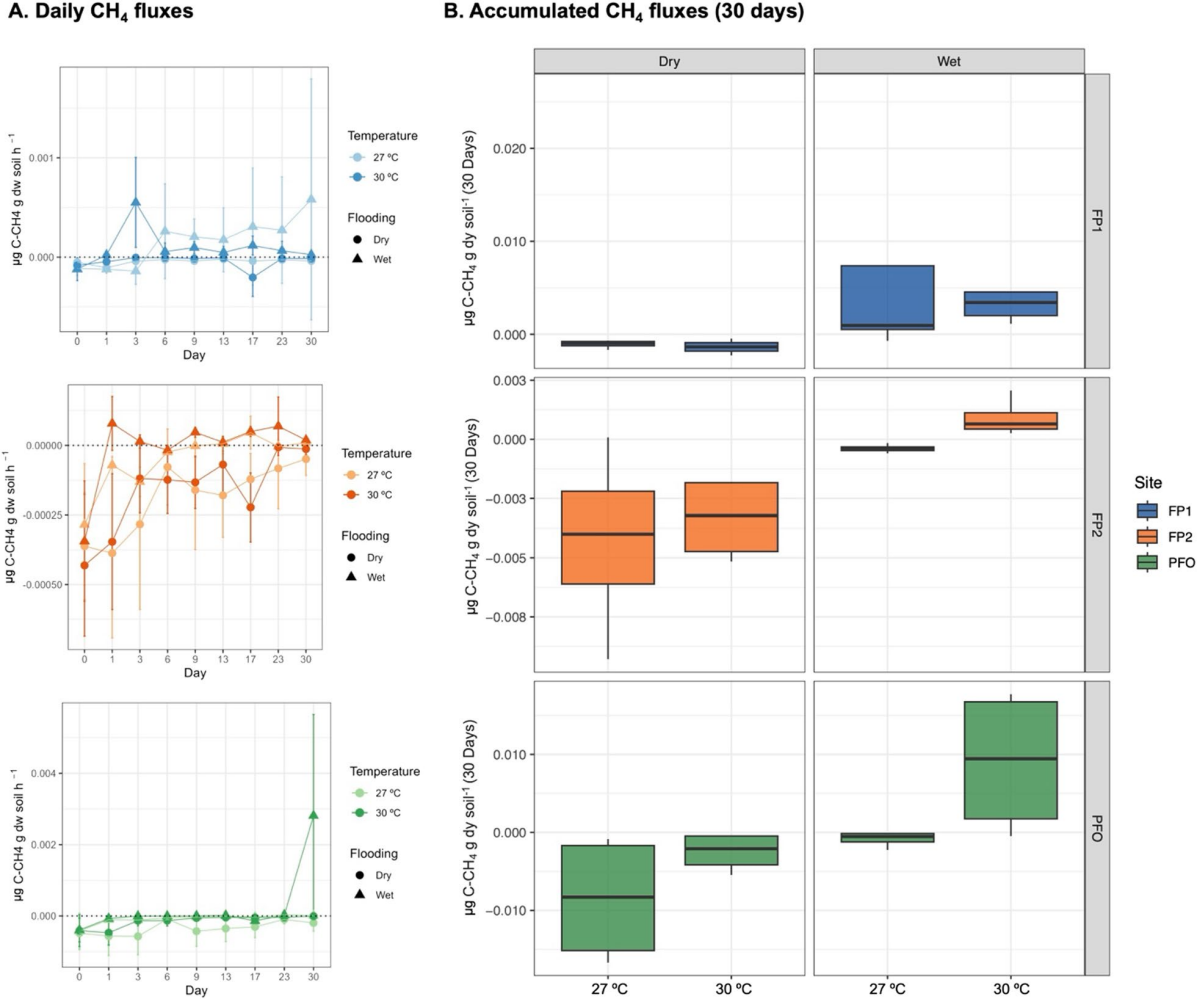
Daily (**a**) and Accumulated (**b**) CH_4_ fluxes measurements in the microcosm experiment. The floodplain (FP1 and FP2) and upland forest (PFO) soils were submitted to changes in flooding (wet and dry) and temperature (27 °C and 30 °C) conditions

The CH_4_ fluxes varied significantly according to treatment conditions and to sample origin. Within each treatment, high variability across sampling points was observed. The evolution of gas fluxes along the experiment also followed different patterns, indicating individual characteristics of each sampled point in the field (Fig. 2a).

In the floodplains, only flooding significantly affected (p < 0.05) the cumulative CH_4_ flux (Fig. 2b; Table 2). The soils from the FP1 site showed negative (− 0.0004 to − 0.002 µg C–CH_4_ g dw soil) and positive (0.0009 to 0.02 µg C–CH_4_ µg dw soil) cumulative fluxes for dry and flooded conditions, respectively, with no significant influence of the temperature increase. However, the CH_4_ fluxes of FP2 exhibited distinct responses compared to FP1. Under flooded conditions, the microcosm displayed CH_4_ consumption at 27 °C and emission at 30 °C. Under dry conditions, the FP2 soil also showed 22% lower potential for CH_4_ consumption due to a 3 °C increase, although this difference was not statistically different (Table 2). The CH_4_ fluxes of the PFO soil were significantly affected by both temperature and flooding (p < 0.05). Within this upland forest soil, an average decrease of 70% in CH_4_ consumption potential was observed with temperature increase in dry conditions. On the last day of the experiment, the CH_4_ emission from PFO soil reached 0.005 µg C–CH_4_ g dw soil h^−1^ under flooding combined with 30 °C ambient temperature conditions, causing a sharp increase in the CH_4_ cumulative emission (Fig. 2b). Additionally, the zoomed-in graph of days 0 to 23 for the PFO illustrates the emission dynamics leading up to the peak. It shows a consistent trend of increased temperature responses, enhancing CH_4_ emission potential under flooding and reducing CH_4_ consumption potential under dry conditions (Supplementary Figure S5).

**Table 2:** Two-way ANOVA of the aligned rank transformed accumulated CH_4_ fluxes data from the floodplain and upland forest soils

Site	Temperature			Flooding			Temperature × flooding		
	df	F	p	df	F	p	df	F	p
FP1	1	0.712	0.415	1	4.302	**0.044**	1	0.495	0.494
FP2	1	1.464	0.249	1	10.745	**0.007**	1	0.000	1.000
PFO	1	5.867	**0.032**	1	12.217	**0.004**	1	0.000	1.000

*FP1* Floodplain 1, *FP2* Floodplain 2, *PFO* Upland Forest, *df* degrees of freedom, *F* F-values

Bold values indicate statistical significance at p-value < 0.05

**Fig. 3:**
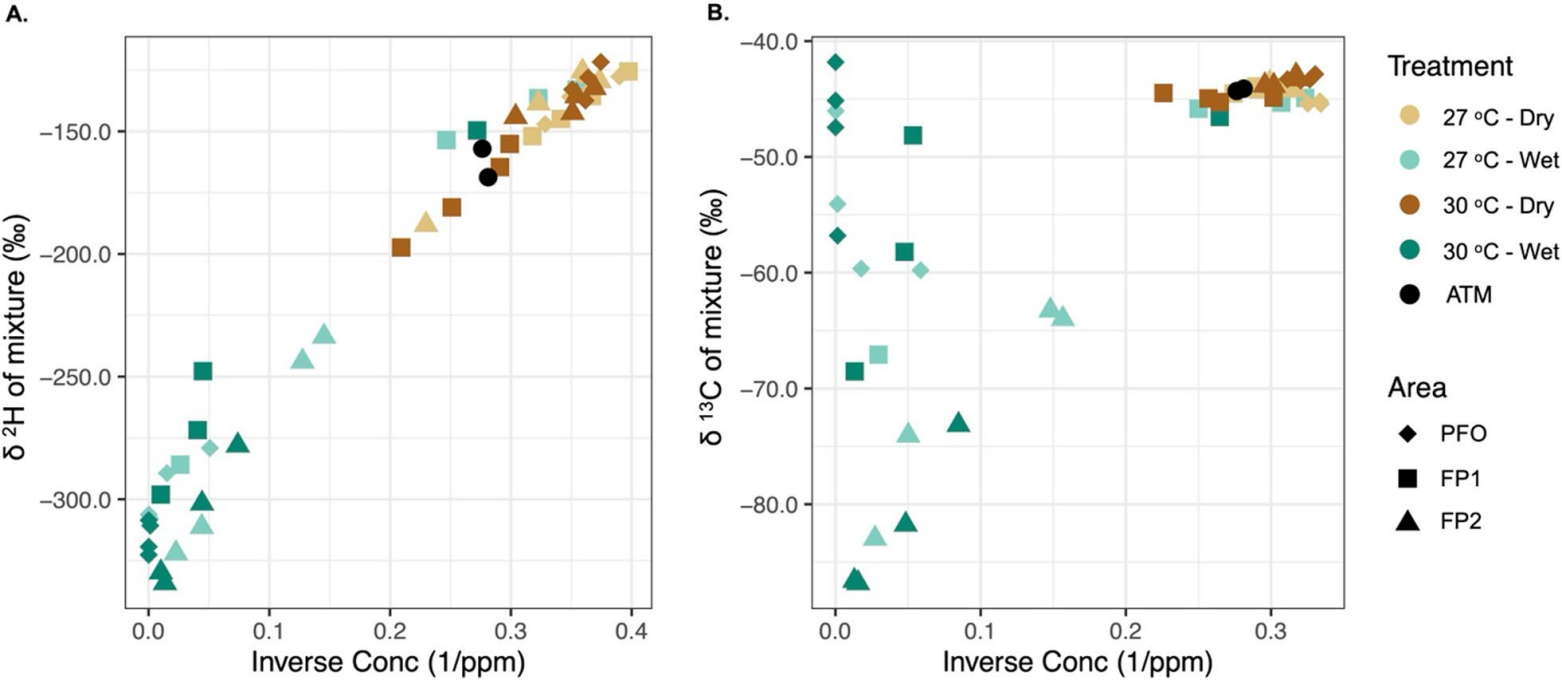
Keeling plot from the ^2^H (**A**) and ^13^C (**B**) isotopic discrimination of CH_4_ samples from the floodplain (FP1 and FP2) and upland forest (PFO) soils under different conditions of flooding (wet and dry) and temperature (27 °C and 30 °C), at the day 30 of the experiment

The results of the isotopic sampling on the last day of the experiment (Day 30) showed a clear effect of flooding on both ^13^C–CH_4_ and ^2^H–CH_4_ (Fig. 3). The reduction in δ^13^C (ranging from − 41.7 to − 103.3 ‰) and δ^2^H (ranging from − 90.4 to 338.6 ‰) under flooding, indicated a prevailing CH_4_ production. When δ^13^C and δ^2^H increased, mostly under dry conditions, they reflected increasing CH_4_ oxidation and, therefore, consumption of this gas in the soil. Interestingly, only a few FP1 samples from wet treatments showed isotope abundance related to CH_4_ oxidation patterns, suggesting the activity of methanotrophic pathways in these samples.

### Diversity and composition of microbial communities

**Fig. 4:**
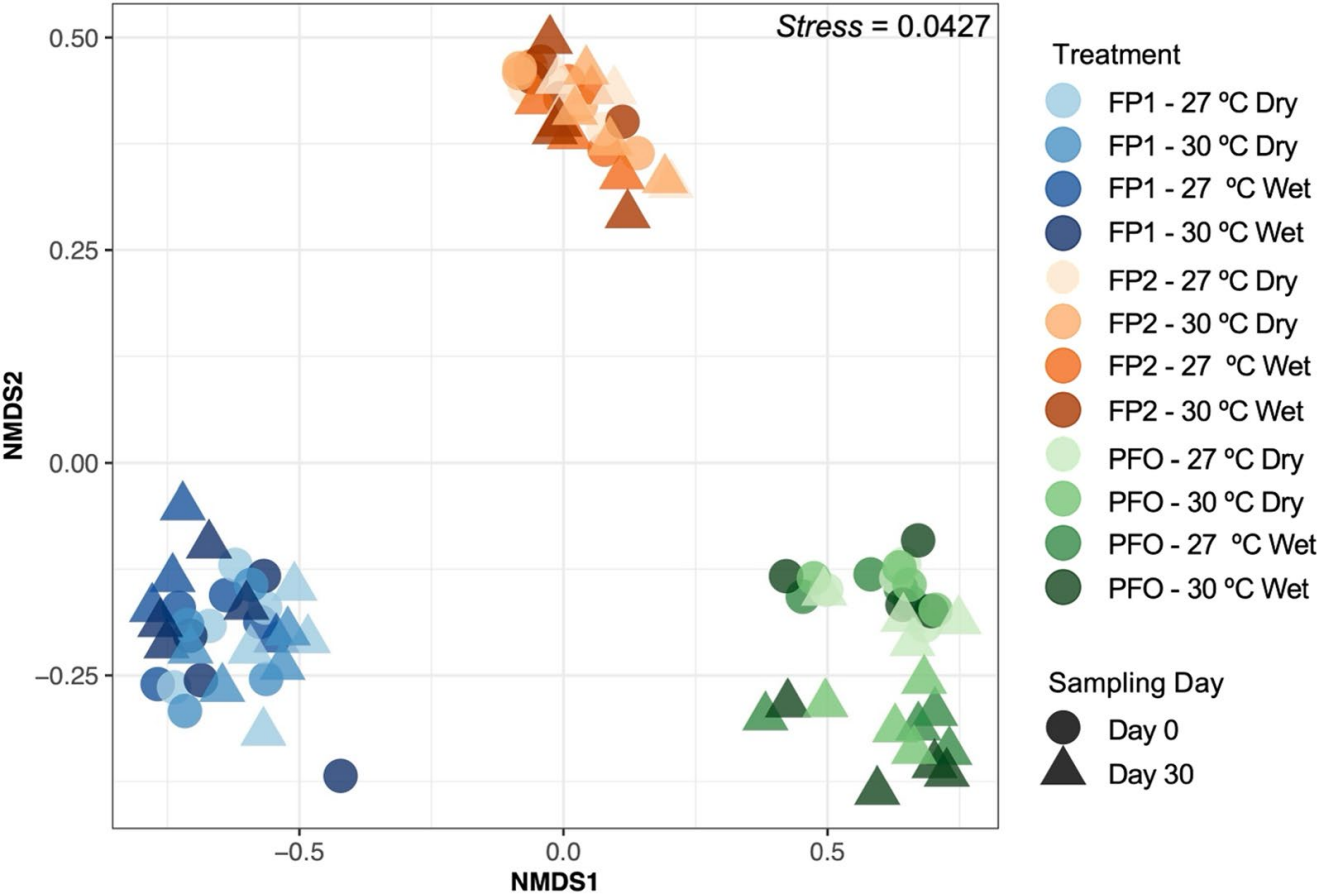
Clustering of the taxonomic structure of the floodplain (FP1 and FP2) and upland forest (PFO) soils under different conditions of flooding (wet and dry) and temperature (27 °C and 30 °C). Plot is based on the non-metric multidimensional scaling (NMDS) using the Bray–Curtis distance index

Soil samples collected from the microcosms on days 0 and 30 were analyzed to capture variations in the diversity and composition of their microbial communities. The sampling day, temperature, flooding, and their interactions were considered as explanatory factors of the dynamics of the total microbial communities, as well as methanogenic and methanotrophic groups. NMDS ordination (Fig. 4) and PERMANOVA analysis (Table 3) indicated that the studied sites harbor distinct microbial communities, reflecting the origin of the samples (R^2^ = 0.808, p < 0.001). Additionally, microbial profiling from floodplains did not show any effect in relation to the treatments applied in the experiment at the DNA level. However, a clear influence of temperature and flooding treatments (where the latter was significant) was observed on upland forest soil samples (Supplementary Figure S6).

**Table 3:** Permutational multivariate analysis of variance of the microbial communities’ taxonomic profile in the floodplain and upland forest soils

Data	FP1			FP2			PFO		
	R^2^	F	p	R^2^	F	p	R^2^	F	p
Sampling day	0.064	1.937	0.066	0.077	2.410	0.054	0.190	7.436	**0.001**
Temperature	0.017	0.518	0.845	0.020	0.632	0.702	0.020	0.780	0.509
Flooding	0.036	1.092	0.317	0.054	1.696	0.129	0.082	3.213	**0.009**
Sampling day × temperature	0.019	0.563	0.809	0.012	0.364	0.947	0.015	0.568	0.716
Sampling day × flooding	0.039	1.187	0.280	0.051	1.581	0.160	0.055	2153	0.066
Temperature × flooding	0.023	0.699	0.646	0.014	0.428	0.887	0.013	0.509	0.772
Sampling day × temperature × flooding	0.013	0.381	0.959	0.012	0.392	0.934	0.013	0.506	0.764

*FP1* Floodplain 1, *FP2* Floodplain 2, *PFO* Upland Forest

Bold values indicate statistical significance at p-value < 0.05. Distance index: Bray–Curtis (amplicon sequencing at genus level)

Regarding microbial profiling, Archaea accounted for 2.1–24.4%, while Bacteria constituted 75.6–97.9% of the community. Within the Archaea domain, Crenarchaeota comprised 0.7–22.8% of the total sequences, followed by Euryarchaeota (0–3.2%), Halobacterota (0–1.6%), and Termoplasmatota (0–1.3%). Notably, FP2 showed a higher relative abundance of archaeal communities, with 11.9–20.7% assigned to the Nitrososphaerace class (Crenarchaeota phylum). On the bacterial side, the most dominant phyla, each exceeding > 5% of the communities, were Proteobacteria, Actinobacteria, and Acidobacteria (Supplementary Figure S7).

**Fig. 5:**
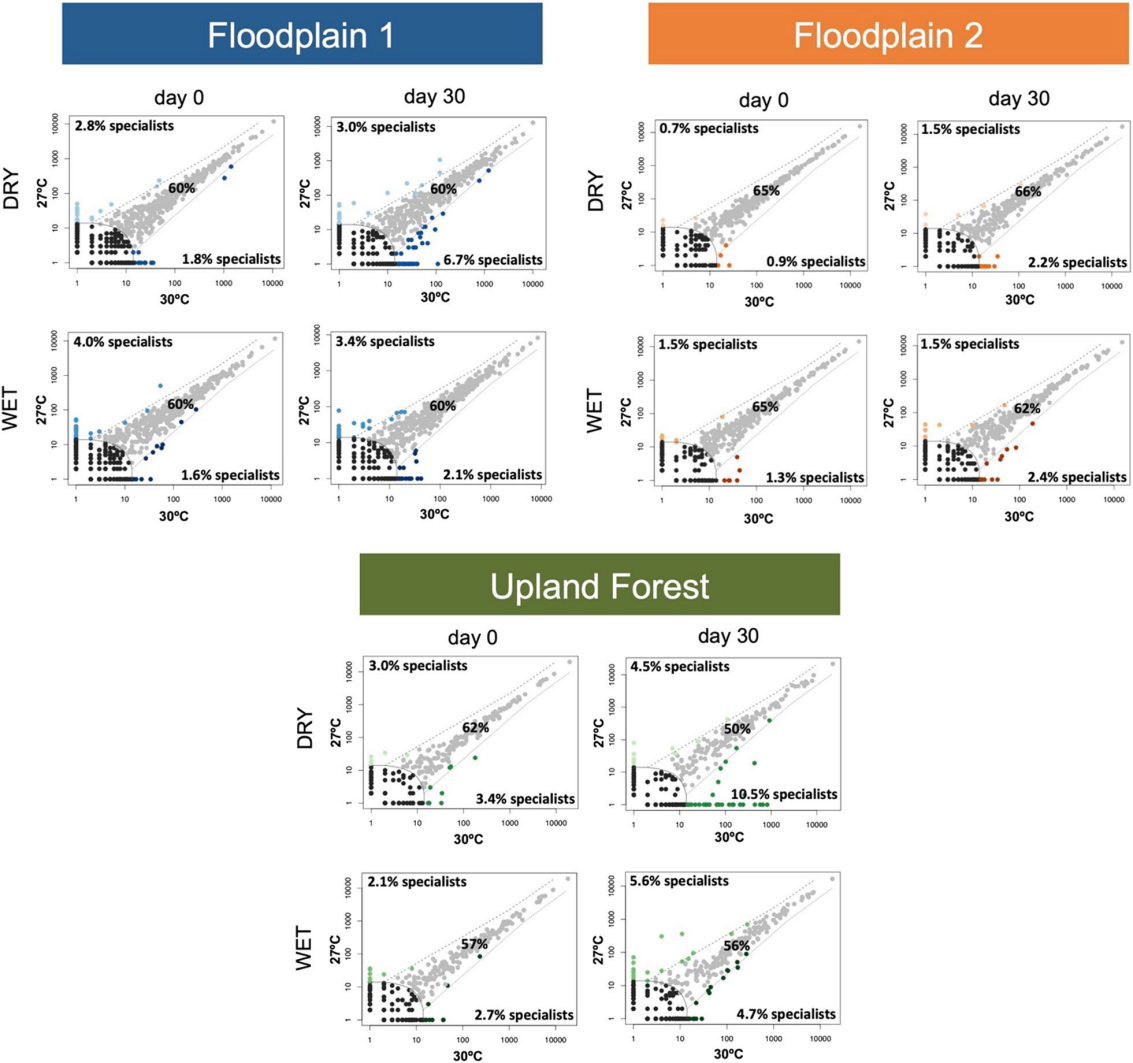
Multinomial species classification method (CLAM) for the niche occupancy test for the microbial communities from the floodplain (FP1 and FP2) and upland forest soils (PFO) under different conditions of flooding (wet and dry) and temperature (27 °C and 30 °C). Days 0 and 30 represents the first and last days of the experiment that the microbial communities where accessed. The proportion of generalists, specialists, and rare is displayed in the graphs. The comparison was made between temperature and flooding conditions for each studied site

We performed a niche occupancy analysis to determine the proportion of microbial generalists and specialists across different treatments for each site (Fig. 5). In general, the microbial generalists remained relatively stable across the floodplains (60% in FP1), with a slight decrease observed in the flooded treatments of FP2 (ranging from 65 to 62%). Our findings also revealed an increase of specialists for habitat resources with increased temperature. Additionally, temperature and flooding conditions strongly affected the generalists/specialists ratio from upland forest soils. Surprisingly, elevating the temperature during 30 days in the PFO under dry conditions led to a selection of organisms strictly related to the local environment, as the abundance of microorganisms identified as generalists was reduced by 12% (from 62 to 50%), while specialists increased from 3.4 to 10.5%.

### Methanogenic and methanotrophic relative abundance

**Fig. 6:**
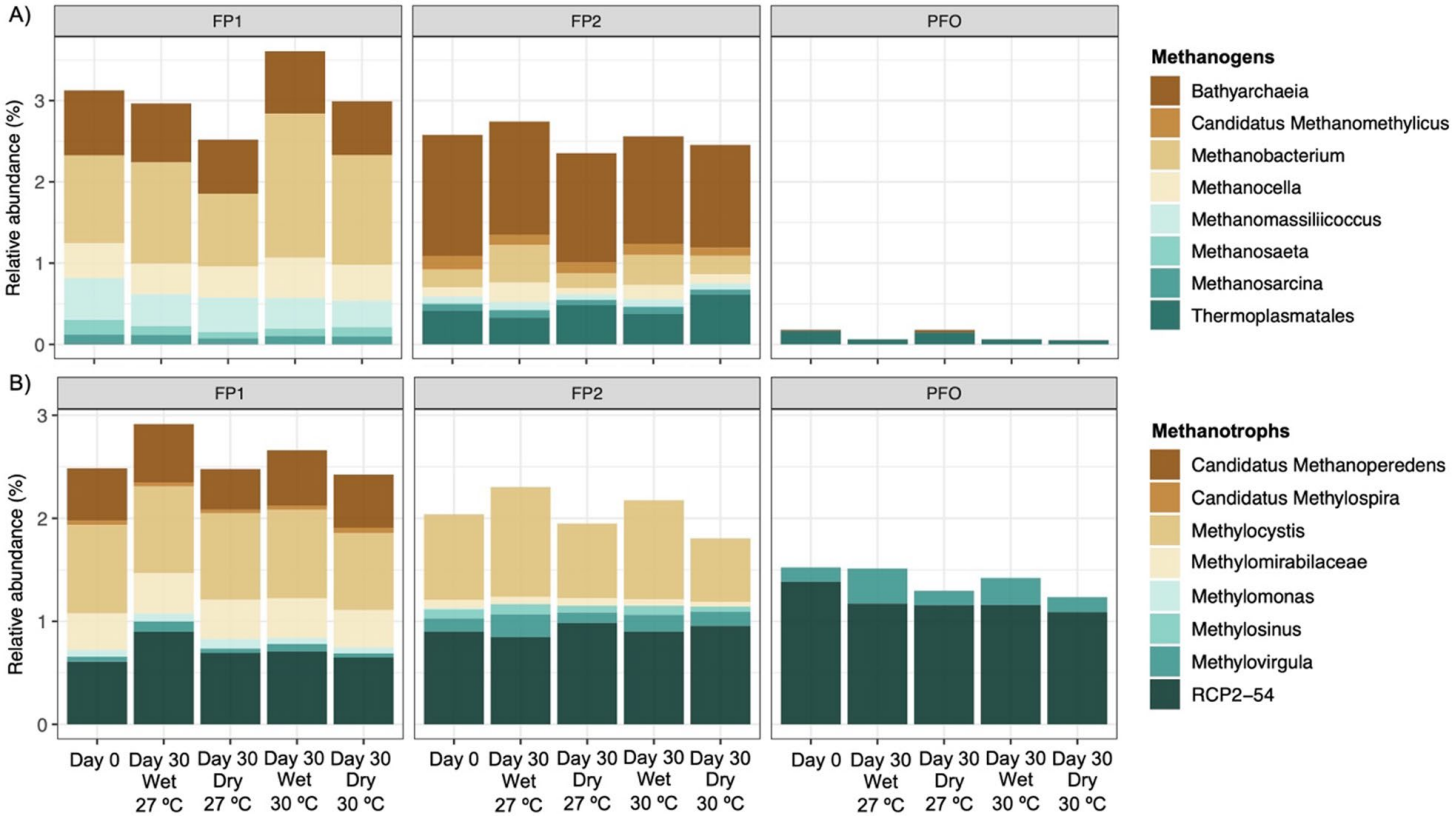
Relative abundance (means per treatment) of the CH_4_-cycling taxa in the floodplain (FP1 and FP2) and upland forest soils (PFO) under different conditions of flooding (wet and dry) and temperature (27 °C and 30 °C). Days 0 and 30 represents the first and last days of the experiment that the microbial communities where accessed

We examined the 16S rRNA gene sequence data to identify taxa with a reported potential role in CH_4_ production or consumption, namely methanogens and methanotrophs, respectively. The overall relative abundance of methanogenic archaea in the floodplain and upland forest soils varied significantly across sites and treatments (Fig. 6a; Table 4; Supplementary Table S3). The methanogenic microbial community reached up to 5.4% (average of 2.6%) of the microbial communities in floodplains, being represented by *Methanobacterium*, *Candidatus Methanomethylicus, Methanobacteria, Methanocella, Methanomassiliicoccus, Methanosaeta, Methanosarcina,* Bathyarchaeia, and Thermoplasmatales. By contrast, potential methanogens comprised less than 0.3% of upland forest soil microbes, which were represented only by the classes Thermoplasmatales and Bathyarchaeia.

**Table 4:** Three-way ANOVA of the aligned rank transformed sequencing (relative abundance of the total methanogens and methanotrophs) and qPCR (16S rRNA Archaea and Bacteria, *mcr*A and *pmo*A) data from the floodplain and upland forest soils

Data	Sampling Day			Temperature			Flooding		
	df	F	p	df	F	p	df	F	p
*FP1*									
Total methanogens (%)	1	0.190	0.667	1	1.135	0.297	1	1.199	0.284
Total methanotrophs (%)	1	0.532	0.473	1	0.030	0.863	1	4.866	**0.037**
16S rRNA Archaea	1	2.862	0.103	1	5.322	**0.030**	1	1.352	0.256
16S rRNA Bacteria	1	61.036	** < 0.001**	1	5.184	**0.032**	1	0.906	0.351
*mcr*A	1	13.355	**0.001**	1	17.711	** < 0.001**	1	6.513	**0.018**
*pmo*A	1	12.378	**0.002**	1	0.191	0.666	1	5.206	**0.032**
*FP2*									
Total methanogens (%)	1	0.288	0.596	1	0.028	0.869	1	0.010	0.921
Total methanotrophs (%)	1	0.005	0.947	1	2.633	0.118	1	6.158	**0.020**
16S rRNA Archaea	1	12.792	**0.002**	1	7.040	**0.014**	1	1.154	0.293
16S rRNA Bacteria	1	19.874	** < 0.001**	1	27.360	** < 0.001**	1	0.001	0.973
*mcr*A	1	31.623	** < 0.001**	1	11.936	**0.002**	1	5.665	**0.026**
*pmo*A	1	0.071	0.792	1	0.039	0.844	1	3.554	**0.042**
*PFO*									
Total methanogens (%)	1	10.064	**0.004**	1	4.188	0.052	1	2.019	0.168
Total methanotrophs (%)	1	0.065	0.427	1	2.026	0.150	1	0.179	0.675
16S rRNA Archaea	1	35.322	** < 0.001**	1	2.144	0.156	1	7.706	**0.010**
16S rRNA Bacteria	1	60.655	** < 0.001**	1	5.447	**0.028**	1	0.801	0.380
*mcr*A	1	37.277	** < 0.001**	1	35.485	** < 0.001**	1	20.299	** < 0.001**
*pmo*A	1	1.358	0.255	1	2.598	0.120	1	6.785	**0.016**

Data	Sampling Day × Temperature			Sampling Day × Flooding			Temperature × Flooding			Sampling Day × Temperature × Flooding		
	df	F	p	df	F	p	df	F	p	df	F	p
*FP1*												
Total methanogens (%)	1	0.004	0.947	1	0.287	0.597	1	0.090	0.597	1	0.001	0.974
Total methanotrophs (%)	1	1.415	0.246	1	2.039	0.166	1	0.268	0.610	1	0.394	0.536
16S rRNA Archaea	1	14.233	** < 0.001**	1	3.455	0.075	1	0.135	0.716	1	1.614	0.216
16S rRNA Bacteria	1	73.691	** < 0.001**	1	1.118	0.301	1	0.545	0.507	1	0.112	0.741
*mcr*A	1	11.087	**0.003**	1	2.286	0.144	1	0.690	0.414	1	2.068	0.163
*pmo*A	1	0.449	0.509	1	0.112	0.741	1	1.740	0.200	1	0.028	0.869
*FP2*												
Total methanogens (%)	1	0.406	0.530	1	2.124	0.158	1	0.973	0.334	1	0.161	0.692
Total methanotrophs (%)	1	0.072	0.791	1	5.880	**0.023**	1	0.162	0.690	1	0.010	0.921
16S rRNA Archaea	1	32.072	** < 0.001**	1	0.337	0.569	1	0.337	0.567	1	0.923	0.346
16S rRNA Bacteria	1	11.120	**0.002**	1	0.295	0.592	1	0.010	0.920	1	0.374	0.546
*mcr*A	1	18.540	** < 0.001**	1	0.010	0.921	1	0.499	0.487	1	0.090	0.760
*pmo*A	1	0.090	0.767	1	0.018	0.895	1	0.288	0.597	1	0.599	0.447
*PFO*												
Total methanogens (%)	1	1.327	0.261	1	0.552	0.465	1	0.160	0.695	1	5.843	**0.024**
Total methanotrophs (%)	1	0.142	0.710	1	0.002	0.876	1	0.000	0.993	1	0.106	0.748
16S rRNA Archaea	1	0.040	0.844	1	6.016	**0.022**	1	7.332	**0.012**	1	8.247	**0.008**
16S rRNA Bacteria	1	59.290	< 0.001	1	0.116	0.736	1	0.094	0.761	1	0.116	0.736
*mcr*A	1	34.780	** < 0.001**	1	20.299	** < 0.001**	1	20.208	** < 0.001**	1	20.229	** < 0.001**
*pmo*A	1	2.350	0.138	1	1.044	0.317	1	1.188	0.287	1	1.622	0.215

*FP1* Floodplain 1, *FP2* Floodplain 2, *PFO* Upland Forest, *df* degrees of freedom, *F* F-values

Bold values indicate statistical significance at p-value < 0.05

The relative abundance of methanotrophs reached up to 3.8% (average of 2.2%) in the floodplains, while it was below 2.4% in the upland forest soils (Fig. 6b; Table 4; Supplementary Table S3). *Methylocystis* was the most dominant methanotrophic genus in the floodplains. Moreover, *Candidatus Methanoperedens, Candidatus Methylospira,* Methylomirabilaceae*, Methylomonas, Methylosinus, Methylovirgula*, and RCP2-54, were also detected. On the other hand, only *Methylovirgula* and RCP2-54 were identified in the upland forest soils.

### Total abundance of archaeal, bacterial, and methane-related microbes

**Fig. 7:**
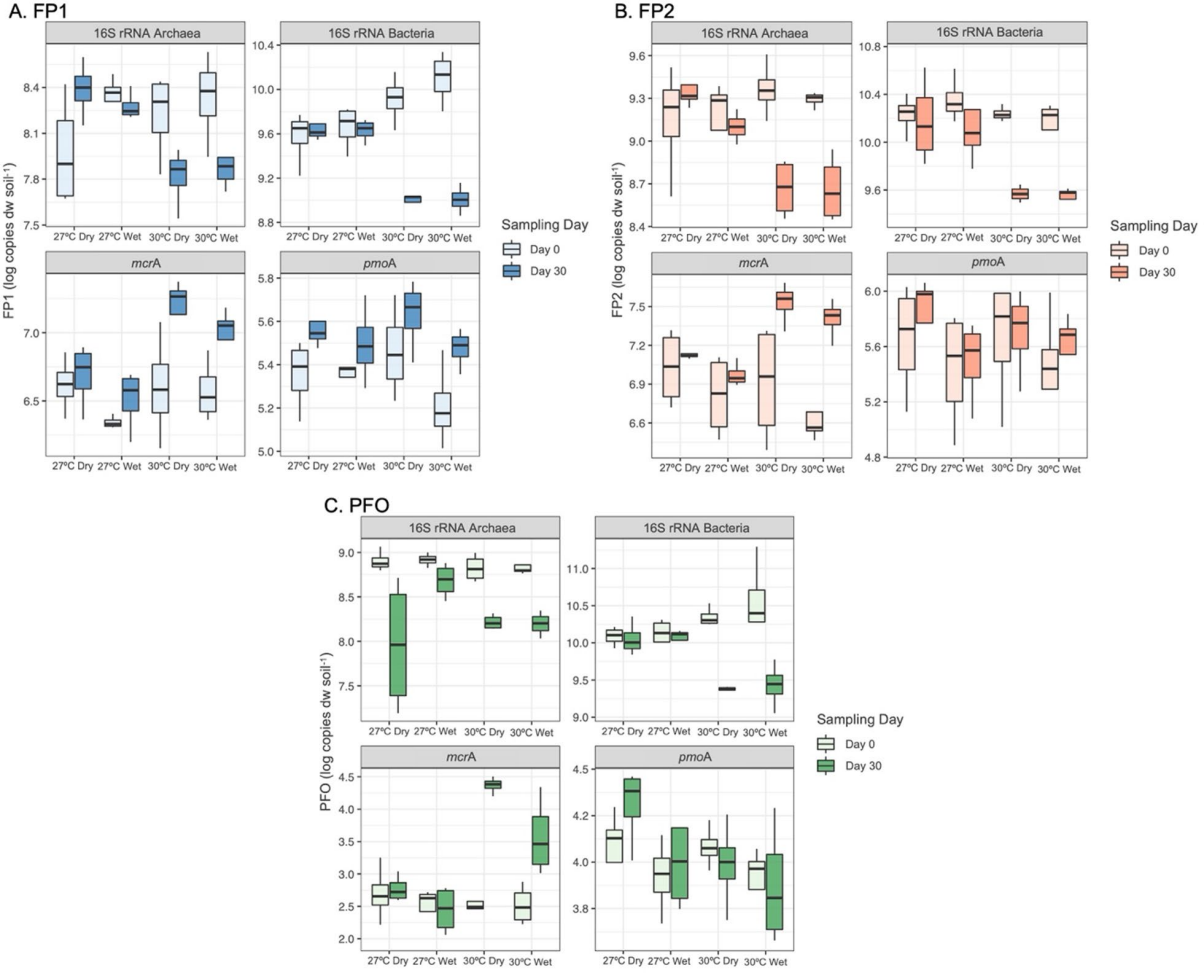
Number of copies per gram of dry weight (log copies dw soil^−1^) of 16S rRNA Archaea, 16S rRNA Bacteria, *mcr*A and *pmo*A genes in the floodplain (FP1 and FP2) and upland forest soils (PFO) under different conditions of flooding (wet and dry) and temperature (27 °C and 30 °C). Days 0 and 30 represents the first and last days of the experiment that the microbial community’s abundances were accessed

By using the qPCR technique, it was possible to detect and quantify the archaeal and bacterial 16S rRNA genes, as well as functional marker genes related to the CH_4_ cycle, *i.e.*, *mcr*A for methanogenic archaea and *pmo*A for methanotrophic bacteria (Fig. 7; Table 4). The standard curves generated for the quantification of all genes showed correlation coefficients above r^2^ = 0.98 and efficiencies between 92 and 108%. Also, sample melting curves showed a single peak that were matching with the standard curves.

In all treatments, the quantification of the archaeal 16S rRNA ranged from 10^7^ to 10^9^ copies/g dry soil^−1^, while the abundance of the bacterial 16S rRNA ranged from 10^9^ to 10^11^ copies/g dry soil^−1^. Interestingly, only the increased temperature significantly affected the 16S rRNA quantifications (p < 0.05). For instance, a clear decrease in the abundance of total microbial community was observed across all three studied sites in the treatments at 30 °C, with more pronounced effects on the Bacterial domain.

The quantification of the *mcr*A gene varied from 10^6^ to 10^7^ copies/g dry soil^−1^ in FP1 and FP2 soils, while in PFO, it varied from 10^0^ to 10^4^ copies/g dry soil^−1^. The sampling day, temperature, flooding, and the interaction of sampling day and temperature factors significantly affected (p < 0.05) the abundance of this gene in floodplains. In the PFO, all factors, including the triple interaction sampling day × temperature × flooding, significantly influenced (p < 0.05) the *mcr*A gene abundance. All areas showed a significant increase in *mcr*A with increasing temperature, regardless of the flooding condition.

The abundance of the *pmo*A gene varied from 10^5^ to 10^6^ copies/g dry soil^−1^ in soils from FP1 and FP2, whereas in PFO soils, its quantities were lower, ranging from 10^3^ to 10^4^ copies/g dry soil^−1^. The total methanotrophic abundance, as indicated by the quantification of the *pmo*A gene, was significantly reduced in response to flooding in FP2 and PFO. Additionally, the period was also significant in FP1 (p < 0.05). Unlike methanogens, the total abundance of methanotrophs did not show a significant influence on temperature in any of the areas.

## Discussion

Many studies have predicted the consequences of climate change in the Amazon basin, including temperature increases and pronounced flooding and drought periods [63, 77], which may also affect the CH_4_ cycle in the region [12]. Our findings reveal that both floodplain and upland forest soils have the potential to act as either sources or sinks of CH_4_, depending on the environmental conditions to which they are subjected. These results align with prior studies conducted in other environments, demonstrating that soil CH_4_ cycling is affected by factors such as flooding and temperature [9, 30, 44, 82].

In our study, we evaluated how these factors and their combinations can affect the dynamics of the CH_4_ fluxes and microbial communities in soils from the Amazon region over a period of 30 days. To achieve this, we used soils from floodplain areas of the Amazon and Tapajós rivers. Despite their proximity, both sites have different origins and limnological characteristics [36]. The chemical characterization of the soils confirmed the influence of the rivers in these areas. The floodplain FP1 had higher quantities of metals, including Fe, Ca, Mg, and Mn, indicating deposition from the Amazon River during the previous wet seasons. By contrast, FP2 displayed elevated levels of OM, N, P, and SO_4_^2−^, reflecting the abundant deposition and accumulation of organic matter from the local forest vegetation, given that the Tapajós River carries low amounts of sediments along its banks. In contrast to floodplain areas, the upland forest soil presented low nutrient and acidic pH levels, as well as high clay content, as previously reported by Gontijo et al. [25].

By subjecting soils to four treatments combining temperature and flooding conditions and closely monitoring CH_4_ fluxes, we made several noteworthy observations. Firstly, in the floodplain soils, mainly the flooding condition exerted a significant influence, leading to a rapid increase in CH_4_ emissions. These results align with the natural wet and dry seasons that these areas experience annually. Conversely, the forest soil exhibited a response to both flooding and temperature conditions. However, the impact on CH_4_ emissions was observed towards the end of the experiment and was only evident under flooding at 30 °C. Recently, Venturini et al. [82] observed a similar pattern CH_4_ emissions from Amazon upland forest soils under saturated conditions (100% moisture at field capacity) after a 30-day incubation period. Here, our results suggests that a combination of factors, including prolonged flooding and elevated temperature, could play a role in influencing CH_4_ dynamics in a consistently well-drained forest soil. These findings highlight the complex interplay between environmental conditions and CH_4_ emissions in both floodplain and forest soils, underscoring the need to consider multiple factors in parallel when assessing the potential for CH_4_ release from different soil types. Hernández et al. [30] reported that the response time for CH_4_ production by the microbial community under anaerobic conditions depends on the flooding history of different areas and is faster in seasonal floodplains when compared to upland areas. Furthermore, according to Scavino et al. [74], in soils that do not have the influence of seasonal inundation, CH_4_ production only starts after a long period of flooding, as the microbial community requires adaptation to a new environmental condition.

Interestingly, we observed a decrease in the total abundance of Bacteria and Archaea at all three sites (based on the qPCR of 16S rRNA genes) in response to temperature, mainly for Bacteria. While it is acknowledged that microbial responses to climate change vary depending on the ecosystem [59], it is noteworthy that the reaction of microbial communities to warming is more intricate than anticipated. This complexity is influenced by various factors including initial conditions, cumulative effects of climate change, seasonality, and soil characteristics [71, 90].

Our 16S rRNA sequencing data indicated that the composition of the microbial communities is clustered mainly by site. The microbial community structures from both floodplains did not show any changes in response to flooding or temperature, while a clear effect of both factors (which was significant for flooding), was observed in the upland forest soils. On short time scales, shifts in environmental factors may impact microbial communities in terms of sensitivity (changes in composition), resistance (no change in composition), and functional redundancy (changes in composition with unaltered functions) [76]. Microbial communities are also recognized for their remarkable metabolic flexibility and physiological adaptations, which enable them to persist in the face of changing conditions [56, 94]. Furthermore, the periodic flooding events may favor microbial taxa adapted for environmental oscillations in Amazonian floodplains [25]. Therefore, microbial communities observed in floodplain soils may be potentially more resistant to temperature increases, while microbial communities from upland forest soils could be more sensitive to the predicted climate change effects tested in this study.

Niche occupancy analysis demonstrated that the 30 °C treatments created favorable conditions for microbial specialists in the studied sites, with stronger effects on PFO. In addition, the increased temperature in combination with dry conditions also resulted in an increase in the abundance of specialists in the upland forest. Microbial specialists are remarkably responsive to environmental disturbances, including soil chemical properties, flooding, and temperature [55, 66, 85]. Specialists’ microbes are indicator species that exhibit distinct ecological preferences, making them highly specific to particular habitats [50]. A well-established ecological concept suggests that the contrasting characteristics of specialists and generalists stem from differences in their resource utilization strategies [91]. Frequent fluctuations in environmental conditions can foster community-level functional resilience to future climate change by promoting an increase in the diversity of taxa with specific physiologies, particularly specialists [28].

Among the methanogens, *Methanobacterium* was the dominant genus in FP1. This group is widely distributed in anaerobic environments across the globe and is known to produce CH_4_ mainly through the hydrogenotrophic pathway, as well as *Methanocella* [17]. The class Bathyarchaeia, which also presented high abundance in FP1 and was dominant in FP2, may have a potential function in processes related to methanogenesis [18], anaerobic degradation of organic matter, and acetogenesis [29, 51]. However, since there is no isolate of Bathyarchaeia to date, the demonstration of these physiological capabilities is still missing. The acetoclastic methanogens *Methanosarcina* and *Methanosaeta* [58], accounted only for a small fraction of the methanogens. Furthermore, *Methanosarcina* has a versatile metabolism that prefers hydrogen, methanol, and methylamine to acetate [86]. We also identified the methylotrophic and hydrogen‐dependent methanogen belonging to the genus *Methanomassiliicoccus* [61], which was part of the dominant groups only in FP1. Although members of the order Thermopasmatales, closely related to *Methanomassiliicoccus*, are also traditionally known as methanogenic, Zinke et al. [98] suggested, based on metagenome-assembled genomes (MAGs), that taxa belonging to this family may not have methanogenic pathways. Instead, the Thermopasmatales MAGs contain genes potentially related to sulfur, nitrogen, and hydrogen metabolisms.

Regardless of the flooding condition, the abundance of the *mcr*A gene significantly increased with increased temperature in all areas. Previously, it has been reported that the methanogenic community remains stable even with the seasonality of dry and flood conditions in rice cultivation areas [47] and in Amazonian floodplains [25]. According to Liu et al. [45], temperature can directly affect the abundance and structure of the methanogenic population. In our experiment, there is evidence that even a modest increase of 3 °C disrupts the stability of the system, effectively fostering the potential for higher methanogenic activity in both floodplains and upland forest soils. Concurrently, this temperature increase demonstrated a reduction in the potential for CH_4_ sink function during dry conditions in PFO. These findings underscore the sensitivity of these ecosystems to temperature changes and highlight the potential for shifts in CH_4_ dynamics, which may have significant implications for mitigating greenhouse gas emissions.

Regarding the methanotrophs, Type II methanotrophs (including *Methylocystis* and *Methylosinus)* account for a large fraction of the methanotrophic communities in the floodplains. These organisms are known to endure fluctuations in the environment, such as variable O_2_ and CH_4_ availability [40]. Recently, Gontijo et al. [24] reported a MAG of *Methylocystis* from Amazonian floodplains. The authors found that this dominant aerobic methanotroph possesses unique genes related to nitrogen metabolism and cell motility, which may contribute to the niche occupancy of this organism in this environment. On the other hand, the Type I methanotrophs *Candidatus* Methylospira and *Methylomonas* were found only in FP1, comprising a small fraction of the methanotrophic community from this site. In general, members of this group are very responsive to high substrate availability [32] and may have their abundance reduced quickly under O_2_ limitation or environmental disturbance [40]. In PFO, only *Methylovirgula* and RCP2-54 (both also detected in the floodplains) were identified. From *Methylovirgula*, traditionally known as methylotroph, a new methanotrophic taxon was recently isolated from a Korean wetland [27]. The authors reported that this *Methylovirgula* possesses the capabilities to aerobically oxidize both CH_4_ and reduced sulfur compounds for growth. Lastly, the poorly characterized RCP2-54 phylum, also classified as the Binatota phylum by the GTDB-tk, have been recently suggested to be involved in CH_4_ oxidation [60]. Recently, Venturini et al. [83] also recovered a Binatota MAG from Amazonian pasture soils with the pMMO operon. This and other yet to be studied taxa could have played a role in mitigating the CH_4_ emissions in the Amazonian region. However, while suggestive, the actual demonstration of these physiological capabilities remains elusive, underscoring the need for further studies to elucidate this potential contribution.

From the isotopic discrimination data, assessed on the last day of the experiment, we found a clear difference between methanogenic and methanotrophic predominant pathways in response to flooding. While the methanotrophic activity resulted in enriched values of δ^13^C and δ^2^H (the heavier isotopes), based on the microbial preference to oxidize lighter molecules, the process of methanogenesis results in the lowest values of δ^13^C and δ^2^H [52]. It is suggested that the CH_4_ produced by the hydrogenotrophic pathway has lower δ^13^C and higher δ^2^H (δ^13^C =  − 110 to − 60‰ and δ^2^H =  − 250 to − 170‰) when compared to CH_4_ produced by the acetoclastic pathway (δ^13^C =  − 60%o to − 50‰ and δ^2^H =  − 400‰ to − 250‰) [10, 87]. In our study, an identification of the dominant methanogenic pathway was not possible, indicating that both hydrogenotrophic and acetoclastic pathways may be active simultaneously. Furthermore, the isotopic data clustered the floodplains apart from each other, mainly regarding δ^13^C, suggesting that these pathways may be active at different weights for each floodplain. Interestingly, some samples from FP1 under flooding conditions presented a CH_4_ oxidation pattern, which may be an indication of both aerobic and anaerobic CH_4_ oxidation activity [5, 25, 35].

In FP1, we also detected both archaeal and bacterial taxa with the reported capability to carry out anaerobic CH_4_ oxidation, which was previously reported by Gontijo et al. [25]. The archaeal genus *Candidatus* Methanoperedens has the capability to carry out anaerobic methanotrophy in consortia with sulfate‐reducing bacteria [80] or using the nitrate-dependent reverse methanogenesis [92]. It has been demonstrated that this genus may perform anaerobic CH_4_ oxidation coupled with the reduction of metals such as Fe(III) and Mn(IV) [22, 43]. In fact, we detected this genus only in FP1, which were also the floodplains with the highest quantities of those metals due to the influence of the Amazonas River. Another group, the bacterial order Methylomirabilales (formerly NC10 phylum), comprises taxa with the capability to oxidize CH_4_ under anaerobic conditions using nitrite as an electron acceptor [16, 65]. Moreover, Bento et al. [5] recently reported the role of NC10 in anaerobic CH_4_ oxidation in Amazonian floodplains.

Our findings underscore the potential role of CH_4_ oxidation pathways in mitigating the rising trend of CH_4_ emissions associated with climate change. Nevertheless, further experimental and field investigations are required to validate these assertions. Future studies could focus on identifying the factors influencing the efficiency of aerobic and anaerobic CH_4_ oxidation in floodplain ecosystems and quantifying its contribution to overall CH_4_ dynamics. Assessing the long-term stability and resilience of these processes in the face of environmental changes would provide valuable insights for understanding their potential role in climate change mitigation.

## Limitations and future research

Our study provides new insights into how rising temperatures in both flooded and non-flooded conditions affect CH_4_ fluxes and microbial communities in Amazon floodplain and upland forest soils. While our comparative approach is robust, the study was conducted in laboratory-controlled conditions with deformed soil samples. Altering soil structure changes aeration and influences CH_4_ fluxes [97], and the field climatic conditions of our study sites vary frequently and dynamically [20], both affecting the magnitude of soil microbial community activity [13]. Furthermore, although flooding in upland forests is currently unlikely, extreme climate scenarios are unpredictable [21]. Thus, our numerical results should not be directly extrapolated to real field conditions, though they indicate the direction of process dynamics. It is important to note that incubating intact soil cores and conducting field ecosystem manipulations would provide a more accurate representation of field responses. Therefore, although very challenging, we advocate using our approach to delineate future in situ assessments in the Amazon. This would involve dynamically increasing the temperature (e.g., by 3 °C) based on real-time weather while maintaining soil structure and other environmental variables.

Lastly, floodplain microbes are likely more adapted to changes like flooding [25]. However, DNA persistence may limit the detection of community changes within a short 30-day period. RNA-based methods would provide a more dynamic view of microbial responses, capturing active microbial processes and rapid community composition changes [70]. Future research should incorporate RNA-based approaches to better capture these dynamics. Combining DNA and RNA analyses could offer a comprehensive understanding of both stable and active microbial communities in floodplain and upland ecosystems under climate change.

## Conclusions

We conclude that soils from floodplains and upland forests in the Amazon region have contrasting responses to increasing temperature and flooding scenarios, particularly in relation to CH_4_ fluxes and microbial community dynamics. The flooding condition, simulating both dry and flooded seasons, emerged as the primary factor influencing the CH_4_ sink and emission potential in the floodplains. By contrast, for the upland forest we demonstrated that temperature also plays a crucial role in shaping the delicate balance between CH_4_ sink and emission, leading to reduce the CH_4_ sink function in dry conditions. Additionally, our findings indicate that higher temperatures also lead to a reduction in the total abundance of Bacteria and Archaea and an increase in the percentage of specialist microbes in the studied sites. Our results underscore the heightened sensitivity of upland soil microbial communities to the climate change effects examined in this study. The floodplains, on the other hand, exhibited a high diversity of methanogens and methanotrophs with different metabolic capabilities. We also observed the influence of temperature on the total abundance of methanogens. The floodplain that presented a higher relative abundance of aerobic and anaerobic methanotrophs also signaled methanotrophic activity by isotopic analysis. Altogether, these findings emphasize the importance of considering both environmental factors when assessing the dynamics of CH_4_ in these distinct ecosystems, contributing to our understanding of the complex interactions between climate, hydrology, and microbial processes in the Amazon region.

### Supplementary Information

Additional file 1.

## Data Availability

The 16S rRNA sequencing dataset generated and analyzed during the current study is available in the NCBI’s Sequence Read Archive (SRA) under the accession number PRJNA1084853.
