# Supplementary material for: Methane-cycling microbial communities from Amazon floodplains and upland forests respond differently to simulated climate change scenarios
Source: Environ Microbiome. 2024 Jul 17;19:48. doi: 10.1186/s40793-024-00596-z (PMC11256501; doi:10.1186/s40793-024-00596-z)
Supplement: Supplementary file 1 — Additional file 1. [file 40793_2024_596_MOESM1_ESM.docx]

**Supplementary material**

**Methane-cycling microbial communities from Amazon floodplains and upland forests respond differently to simulated climate change scenarios**

Júlia B. Gontijo^1,2*^; Fabiana S. Paula^1^; Wanderlei Bieluczyk^1^; Aline G. França^1^; Deisi Navroski^1^; Jéssica A. Mandro^1^; Andressa M. Venturini^3^; Fernanda O. Asselta^1^; Lucas W. Mendes^1^; José M. S. Moura^4^; Marcelo Z. Moreira^1^; Klaus Nüsslein^5^; Brendan J.M. Bohannan^6^; Paul L.E. Bodelier^7^; Jorge L. Mazza Rodrigues^2,8^; Siu M. Tsai^1^

^1^Centro de Energia Nuclear na Agricultura, Universidade de São Paulo, Piracicaba, SP, Brazil

^2^Department of Land, Air and Water Resources, University of California, Davis, CA, USA

^3^Department of Biology, Stanford University, Stanford, CA, USA

^4^Instituto de Formação Interdisciplinar e Intercultural, Universidade Federal do Oeste do Pará, Santarém, PA, Brazil

^5^Department of Microbiology, University of Massachusetts, Amherst, MA, USA

^6^Institute of Ecology and Evolution, Department of Biology, University of Oregon, Eugene, OR, USA

^7^Netherlands Institute of Ecology, NIOO-KNAW, Wageningen, GE, The Netherlands

^8^Environmental Genomics and Systems Biology Division, Lawrence Berkeley National Laboratory, Berkeley, CA, USA

^*^Corresponding author: [jbgontijo@ucdavis.edu](mailto:jbgontijo@ucdavis.edu)

**Supplementary Tables**

**Table S1.** Chemical properties of the floodplain and upland forest soils during wet and dry seasons.

| **Parameter** | **Unit** | **FP1** | | | **FP2** | | | **PFO** | | |
| --- | --- | --- | --- | --- | --- | --- | --- | --- | --- | --- |
| pH (H_2_O) | - | 5,3 | ± | 0,5^a^ | 5 | ± | 0,1^a^ | 4 | ± | 0,1^b^ |
| OM | g/dm^3^ | 10,8 | ± | 3,9^c^ | 503,2 | ± | 27,8^a^ | 47,8 | ± | 11,0^b^ |
| N | mg/Kg | 882 | ± | 106,2^b^ | 11914 | ± | 2526,5^a^ | 2828 | ± | 1165,7^ab^ |
| SO_4_^2-^ | mg/dm^3^ | 8,8 | ± | 2,3^b^ | 18,2 | ± | 3,0^a^ | 11,8 | ± | 1,6^ab^ |
| P | mg/dm^3^ | 7,4 | ± | 2,1^b^ | 13 | ± | 1,6^a^ | 8,8 | ± | 1,3^b^ |
| K | mmolc/dm^3^ | 1,7 | ± | 0,4^a^ | 1,8 | ± | 0,5^a^ | 1,2 | ± | 0,1^a^ |
| Ca | mmolc/dm^3^ | 42,2 | ± | 20,1^a^ | 2,4 | ± | 0,5^b^ | 4,6 | ± | 1,9^b^ |
| Mg | mmolc/dm^3^ | 33,8 | ± | 13,5^a^ | 2,4 | ± | 0,9^b^ | 3,4 | ± | 0,9^b^ |
| Al | mmolc/dm^3^ | 16,4 | ± | 8,8^b^ | 71,6 | ± | 5,6^a^ | 67,8 | ± | 11,1^a^ |
| Cu | mg/dm^3^ | 2,1 | ± | 0,4^a^ | 0,3 | ± | 0,1^b^ | 0,3 | ± | 0,1^b^ |
| Fe | mg/dm^3^ | 232 | ± | 83,5^a^ | 7,4 | ± | 1,9^b^ | 190,2 | ± | 70,6^a^ |
| Zn | mg/dm^3^ | 3,3 | ± | 0,7^a^ | 1,2 | ± | 0,3^b^ | 1,7 | ± | 1,3^ab^ |
| Mn | mg/dm^3^ | 31,2 | ± | 11,9^a^ | 6,3 | ± | 0,5^b^ | 8,5 | ± | 1,7^ab^ |
| B | mg/dm^3^ | 0,2 | ± | 0,1^a^ | 0,4 | ± | 0,1^a^ | 0,3 | ± | 0,1^a^ |
| Clay | g/kg | 308 | ± | 54,5^b^ | 362 | ± | 28,5^b^ | 775 | ± | 59,2^a^ |
| Silt | g/kg | 466 | ± | 60,6^a^ | 284 | ± | 45,6^ab^ | 179 | ± | 62,3^b^ |
| Sand | g/kg | 226 | ± | 111,7^a^ | 354 | ± | 50,8^ab^ | 46 | ± | 15,2^b^ |

pH: hydrogen potential; OM: organic matter; N: nitrogen P: phosphorus, SO_4_^2-^: sulfate; K: potassium; Ca: calcium; Mg: magnesium; Al: aluminium; B: boron; Cu: copper; Fe: iron; Mn manganese; Zn: zinc. Values are presented as means ± standard deviation. Lower-case letters indicate the comparison between sites (Kruskal-Wallis and Dunn Test; p < 0.05). FP1: Floodplain 1; FP2: Floodplain 2; PFO: Upland Forest.

**Table S2.** Results of the envfit analysis - correlation of the soil chemical and physical properties in the floodplain and upland forest soils.

| **Parameter** | **R^2^** | **p-value** |
| --- | --- | --- |
| *Soil chemical and physical properties* | | |
| pH | 0.513 | **0.012** |
| OM | 0.860 | **0.002** |
| N | 0.802 | **0.002** |
| SO_4_^2-^ | 0.724 | **0.002** |
| P | 0.588 | **0.004** |
| K | 0.495 | **0.013** |
| Ca | 0.779 | **0.001** |
| Mg | 0.829 | **0.001** |
| Al | 0.919 | **0.001** |
| Cu | 0.958 | **0.001** |
| Fe | 0.588 | **0.009** |
| Zn | 0.562 | **0.006** |
| Mn | 0.778 | **0.001** |
| B | 0.324 | 0.082 |
| Clay | 0.874 | **0.001** |
| Silt | 0.740 | **0.001** |
| Sand | 0.892 | **0.001** |
| *Factor* | | |
| Site | 0.949 | **0.001** |

pH: hydrogen potential; OM: organic matter; N: nitrogen P: phosphorus, SO_4_^2-^: sulfate; K: potassium; Ca: calcium; Mg: magnesium; Al: aluminium; B: boron; Cu: copper; Fe: iron; Mn manganese; Zn: zinc. Bold values indicate statistical significance at p-value < 0.05.

**Table S3.** Three-way ANOVA of the aligned rank transformed relative abundance of methanogens and methanotrophs from the floodplain and upland forest soils.

| **Data** | **Sampling Day** | | | **Temperature** | | | **Flooding** | | |
| --- | --- | --- | --- | --- | --- | --- | --- | --- | --- |
|  | **gl** | **F** | **p** | **gl** | **F** | **p** | **gl** | **F** | **p** |
| ***Floodplain 1 - FP1*** | | | | | | | | | |
| Bathyarchaeia | 1 | 0.058 | 0.811 | 1 | 0.019 | 0.892 | 1 | 0.042 | 0.838 |
| *Methanobacterium* | 1 | 1.322 | 0.262 | 1 | 2.648 | 0.117 | 1 | 0.617 | 0.440 |
| *Methanocella* | 1 | 0.018 | 0.895 | 1 | 1.623 | 0.215 | 1 | 0.010 | 0.921 |
| *Methanosaeta* | 1 | 25.183 | **<0.001** | 1 | 0.168 | 0.686 | 1 | 0.117 | 0.736 |
| *Methanosarcina* | 1 | 1.923 | 0.178 | 1 | 0.190 | 0.667 | 1 | 0.040 | 0.842 |
| *Methanomassiliicoccus* | 1 | 2.133 | 0.157 | 1 | 0.054 | 0.818 | 1 | 0.219 | 0.644 |
| Thermoplasmatales | 1 | 1.549 | 0.225 | 1 | 0.005 | 0.945 | 1 | 0.229 | 0.637 |
| Candidatus *Methanoperedens* | 1 | 0.000 | 1.000 | 1 | 0.256 | 0.618 | 1 | 0.660 | 0.425 |
| *Methylomirabilia* | 1 | 0.630 | 0.437 | 1 | 0.019 | 0.892 | 1 | 0.630 | 0.435 |
| *Methylocystis* | 1 | 0.500 | 0.486 | 1 | 0.018 | 0.895 | 1 | 0.653 | 0.427 |
| *Methylovirgula* | 1 | 1.619 | 0.215 | 1 | 6.000 | **0.029** | 1 | 6.530 | **0.0174** |
| Candidatus *Methylospira* | 1 | 0.090 | 0.767 | 1 | 0.449 | 0.509 | 1 | 2.345 | 0.139 |
| *Methylomonas* | 1 | 0.404 | 0.501 | 1 | 1.039 | 0.318 | 1 | 0.404 | 0.531 |
| RCP2-54 | 1 | 0.257 | 0.616 | 1 | 0.169 | 0.685 | 1 | 1.012 | 0.324 |
| ***Floodplain 2 - FP2*** | | | | | | | | | |
| Bathyarchaeia | 1 | 1.276 | 0.230 | 1 | 0.004 | 0.947 | 1 | 0.018 | 0.895 |
| Candidatus *Methanomethylicus* | 1 | 1.714 | 0.203 | 1 | 0.004 | 0.843 | 1 | 0.896 | 0.353 |
| *Methanobacterium* | 1 | 5.650 | **0.026** | 1 | 0.054 | 0.818 | 1 | 5.383 | **0.029** |
| *Methanocella* | 1 | 6.932 | **0.015** | 1 | 1.661 | 0.210 | 1 | 17.388 | **<0.001** |
| *Methanosarcina* | 1 | 0.708 | 0.408 | 1 | 0.054 | 0.818 | 1 | 0.135 | 0.717 |
| *Methanomassiliicoccus* | 1 | 0.071 | 0.792 | 1 | 0.452 | 0.508 | 1 | 2.276 | 0.144 |
| Thermoplasmatales | 1 | 0.658 | 0.425 | 1 | 0.714 | 0.406 | 1 | 11.409 | **0.002** |
| *Methylomirabilia* | 1 | 0.717 | 0.406 | 1 | 0.219 | 0.644 | 1 | 0.071 | 0.792 |
| *Methylocystis* | 1 | 0.160 | 0.693 | 1 | 0.501 | 0.486 | 1 | 7.023 | **0.014** |
| *Methylosinus* | 1 | 0.251 | 0.621 | 1 | 0.499 | 0.487 | 1 | 0.964 | 0.336 |
| *Methylovirgula* | 1 | 0.365 | 0.551 | 1 | 0.018 | 0.895 | 1 | 2.281 | 0.144 |
| RCP2-54 | 1 | 0.7152 | 0.406 | 1 | 0.055 | 0.817 | 1 | 1.720 | 0.202 |
| ***Upland forest - PFO*** | | | | | | | | | |
| Bathyarchaeia | 1 | 0.126 | 0.725 | 1 | 0.039 | 0.845 | 1 | 0.136 | 0.716 |
| Thermoplasmatales | 1 | 9.751 | **0.005** | 1 | 3.648 | 0.068 | 1 | 2.408 | 0.134 |
| Methylovirgula | 1 | 3.855 | 0.061 | 1 | 0.213 | 0.648 | 1 | 3.301 | 0.082 |
| RCP2-54 | 1 | 5.698 | **0.025** | 1 | 2.788 | 0.107 | 1 | 0.005 | 0.944 |

df: degrees of freedom, F: F-values. Bold values indicate statistical significance at p-value < 0.05.

**Table S3** **(continuation).** Three-way ANOVA of the aligned rank transformed relative abundance of methanogens and methanotrophs from the floodplain and upland forest soils.

| **Data** | **Sampling Day x Temperature** | | | **Sampling Day x Flooding** | | | **Temperature x Flooding** | | | **Sampling Day x Temperature x Flooding** | | |
| --- | --- | --- | --- | --- | --- | --- | --- | --- | --- | --- | --- | --- |
|  | **gl** | **F** | **p** | **gl** | **F** | **p** | **gl** | **F** | **p** | **gl** | **F** | **p** |
| ***Floodplain 1 - FP1*** | | | | | | | | | | | | |
| Bathyarchaeia | 1 | 0.019 | 0.892 | 1 | 0.303 | 0.587 | 1 | 0.424 | 0.521 | 1 | 0.573 | 0.457 |
| *Methanobacterium* | 1 | 0.338 | 0.566 | 1 | 0.683 | 0.417 | 1 | 0.005 | 0.947 | 1 | 0.005 | 0.764 |
| *Methanocella* | 1 | 0.028 | 0.870 | 1 | 0.405 | 0.531 | 1 | 0.405 | 0.531 | 1 | 1.118 | 0.301 |
| *Methanosaeta* | 1 | 0.679 | 0.418 | 1 | 0.170 | 0.684 | 1 | 0.010 | 0.919 | 1 | 2.261 | 0.146 |
| *Methanosarcina* | 1 | 0.136 | 0.716 | 1 | 3.417 | 0.077 | 1 | 1.271 | 0.271 | 1 | 0.500 | 0.486 |
| *Methanomassiliicoccus* | 1 | 0.027 | 0.869 | 1 | 0.111 | 0.742 | 1 | 0.188 | 0.668 | 1 | 0.001 | 0.973 |
| Thermoplasmatales | 1 | 0.165 | 0.688 | 1 | 0.398 | 0.534 | 1 | 0.686 | 0.416 | 1 | 0.001 | 0.973 |
| Candidatus *Methanoperedens* | 1 | 0.460 | 0.504 | 1 | 1.286 | 0.268 | 1 | 0.165 | 0.688 | 1 | 1.054 | 0.315 |
| *Methylomirabilia* | 1 | 0.004 | 0.947 | 1 | 1.070 | 0.311 | 1 | 0.951 | 0.339 | 1 | 0.231 | 0.635 |
| *Methylocystis* | 1 | 1.056 | 0.314 | 1 | 0.712 | 0.407 | 1 | 0.071 | 0.792 | 1 | 0.457 | 0.506 |
| *Methylovirgula* | 1 | 0.840 | 0.369 | 1 | 1.721 | 0.202 | 1 | 0.018 | 0.895 | 1 | 0.452 | 0.508 |
| Candidatus *Methylospira* | 1 | 0.286 | 0.598 | 1 | 0.832 | 0.371 | 1 | 1.800 | 0.192 | 1 | 0.218 | 0.645 |
| *Methylomonas* | 1 | 0.713 | 0.407 | 1 | 0.071 | 0.793 | 1 | 1.180 | 0.283 | 1 | 2.234 | 0.148 |
| RCP2-54 | 1 | 0.624 | 0.437 | 1 | 0.074 | 0.788 | 1 | 0.166 | 0.687 | 1 | 0.377 | 0.545 |
| ***Floodplain 2 - FP2*** | | | | | | | | | | | | |
| Bathyarchaeia | 1 | 0.219 | 0.644 | 1 | 0.367 | 0.550 | 1 | 0.135 | 0.716 | 1 | 0.251 | 0.621 |
| Candidatus *Methanomethylicus* | 1 | 0.287 | 0.597 | 1 | 1.350 | 2.567 | 1 | 0.010 | 0.921 | 1 | 0.498 | 0.487 |
| *Methanobacterium* | 1 | 0.598 | 0.447 | 1 | 9.714 | **0.005** | 1 | 0.599 | 0.447 | 1 | 0.834 | 0.370 |
| *Methanocella* | 1 | 0.001 | 0.973 | 1 | 18.153 | **<0.001** | 1 | 8.089 | **0.009** | 1 | 1.939 | 0.177 |
| *Methanosarcina* | 1 | 0.160 | 0.692 | 1 | 4.957 | **0.036** | 1 | 0.160 | 0.692 | 1 | 0.286 | 0.598 |
| *Methanomassiliicoccus* | 1 | 0.496 | 0.488 | 1 | 0.219 | 0.644 | 1 | 1.525 | 0.229 | 1 | 0.288 | 0.597 |
| Thermoplasmatales | 1 | 1.916 | 0.179 | 1 | 7.625 | **0.011** | 1 | 0.606 | 0.444 | 1 | 0.090 | 0.767 |
| *Methylomirabilia* | 1 | 0.452 | 0.508 | 1 | 0.071 | 0.792 | 1 | 0.090 | 0.767 | 1 | 0.136 | 0.716 |
| *Methylocystis* | 1 | 0.497 | 0.488 | 1 | 4.756 | **0.039** | 1 | 0.111 | 0.742 | 1 | 0.188 | 0.668 |
| *Methylosinus* | 1 | 0.004 | 0.948 | 1 | 0.071 | 0.793 | 1 | 0.071 | 0.793 | 1 | 0.001 | 0.974 |
| *Methylovirgula* | 1 | 0.251 | 0.621 | 1 | 0.367 | 0.550 | 1 | 0.973 | 0.334 | 1 | 0.253 | 0.619 |
| RCP2-54 | 1 | 0.367 | 0.550 | 1 | 0.716 | 0.406 | 1 | 0.040 | 0.842 | 1 | 0.919 | 0.347 |
| ***Upland forest - PFO*** | | | | | | | | | | | | |
| Bathyarchaeia | 1 | 2.008 | 0.169 | 1 | 0.897 | 0.353 | 1 | 0.030 | 0.863 | 1 | 0.406 | 0.530 |
| Thermoplasmatales | 1 | 1.187 | 0.287 | 1 | 0.688 | 0.415 | 1 | 0.164 | 0.689 | 1 | 4.902 | **0.037** |
| Methylovirgula | 1 | 0.978 | 0.333 | 1 | 0.191 | 0.666 | 1 | 0.378 | 0.544 | 1 | 0.290 | 0.595 |
| RCP2-54 | 1 | 0.049 | 0.827 | 1 | 0.008 | 0.930 | 1 | 0.085 | 0.773 | 1 | 0.373 | 0.547 |

df: degrees of freedom, F: F-values. Bold values indicate statistical significance at p-value < 0.05.

**Supplementary Figures**

**
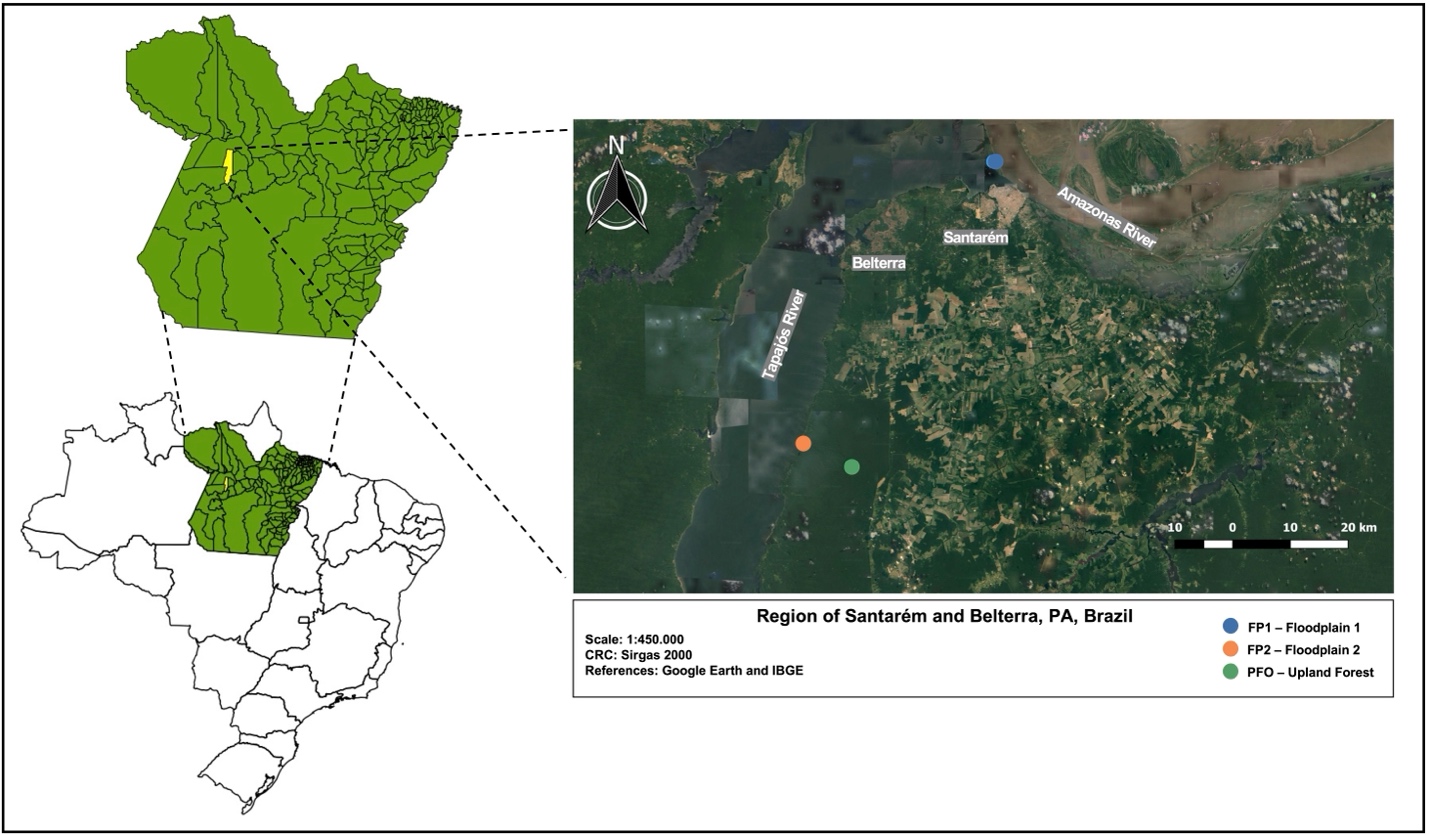
**

**Figure S1.** Location of the studied sites.


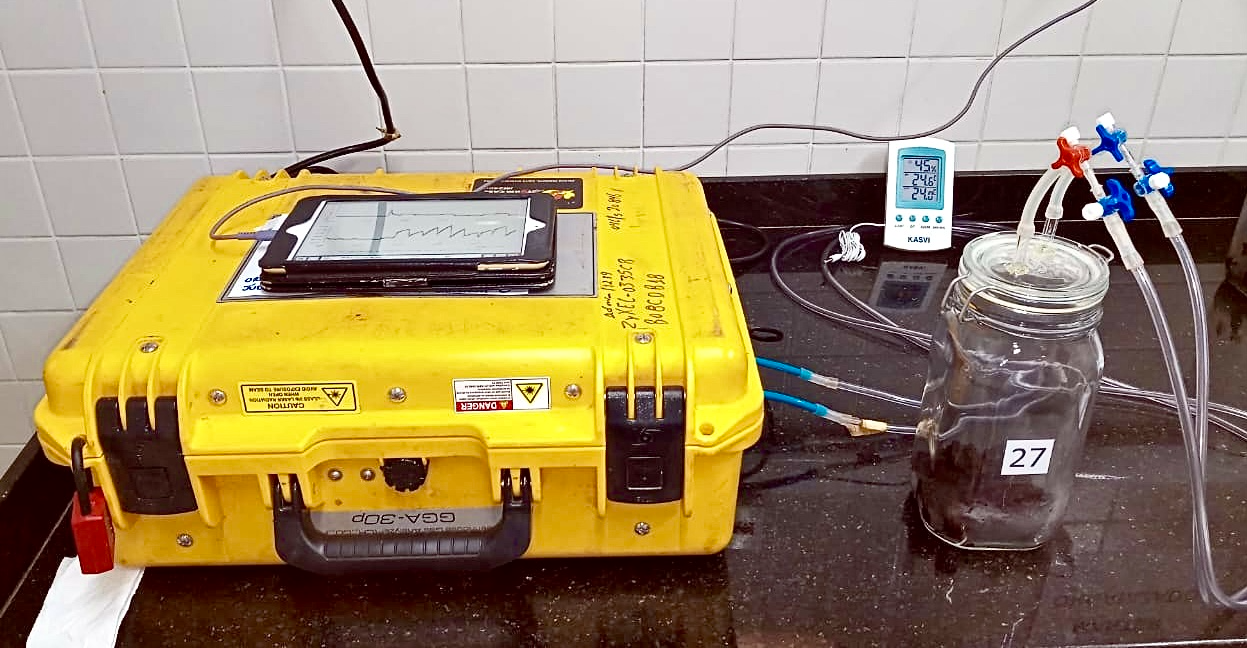


**Figure S2.** Gas measurement using the Ultra-Portable Greenhouse Gas Analyzer (Los Gatos Research, USA).


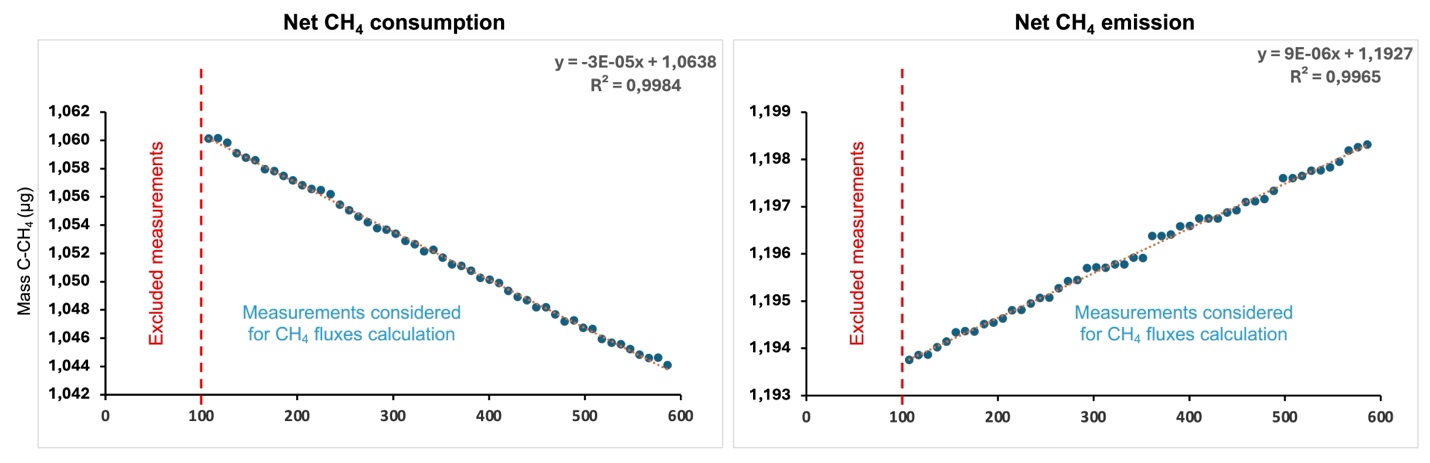


**Figure S3.** Example of the linear regression analysis of CH_4_ concentration measurements during incubation period. The graphs display the CH_4_ concentrations over time, excluding the first 100 seconds to reduce fluctuations. Two examples are provided: one demonstrating CH_4_ consumption and the other showing positive CH_4_ emissions. The high R² values (greater than 0.9) indicate a strong linear relationship, demonstrating the accuracy and reliability of the flux calculations based on these measurements.


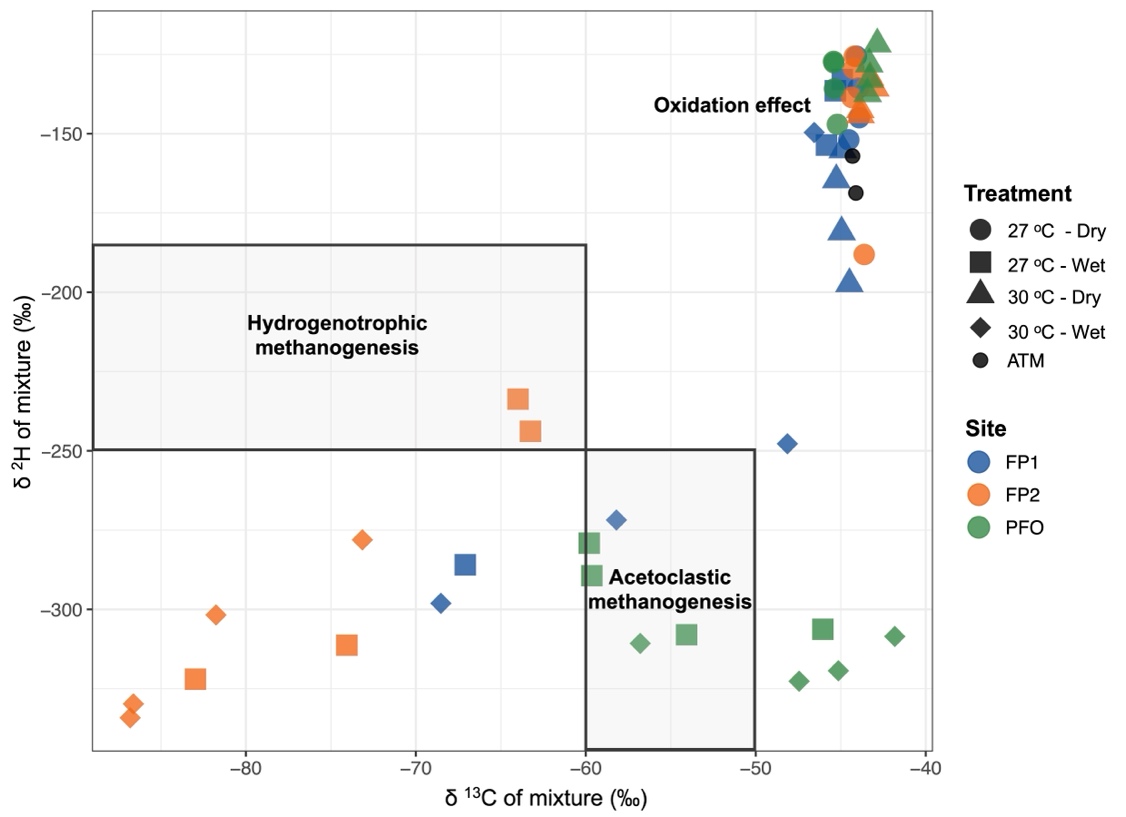


**Figure S4.** Isotopic signatures (δ^13^C and δ^2^H) for CH_4_ production pathways according to Chanton et al. (2005) and Whiticar (1999), from floodplain (FP1 and FP2) and upland forest (PFO) soils samples under different conditions of flooding (wet and dry) and temperature (27 ºC and 30 ºC), at the day 30 of the experiment. The CH_4_ produced by the hydrogenotrophic pathway has lower δ^13^C and higher δ^2^H (δ^13^C = -110‰ to -60‰ and δ^2^H = -250‰ to -170‰) when compared to the CH_4_ produced by the acetoclastic pathway (δ^13^C = -60‰ to -50‰ and δ^2^H = -400‰ to -250‰). Also, methanotrophic activity results in enriched values of δ^13^C and δ^2^H (the heavier isotopes), based on the microbial preference to oxidize lighter molecules.


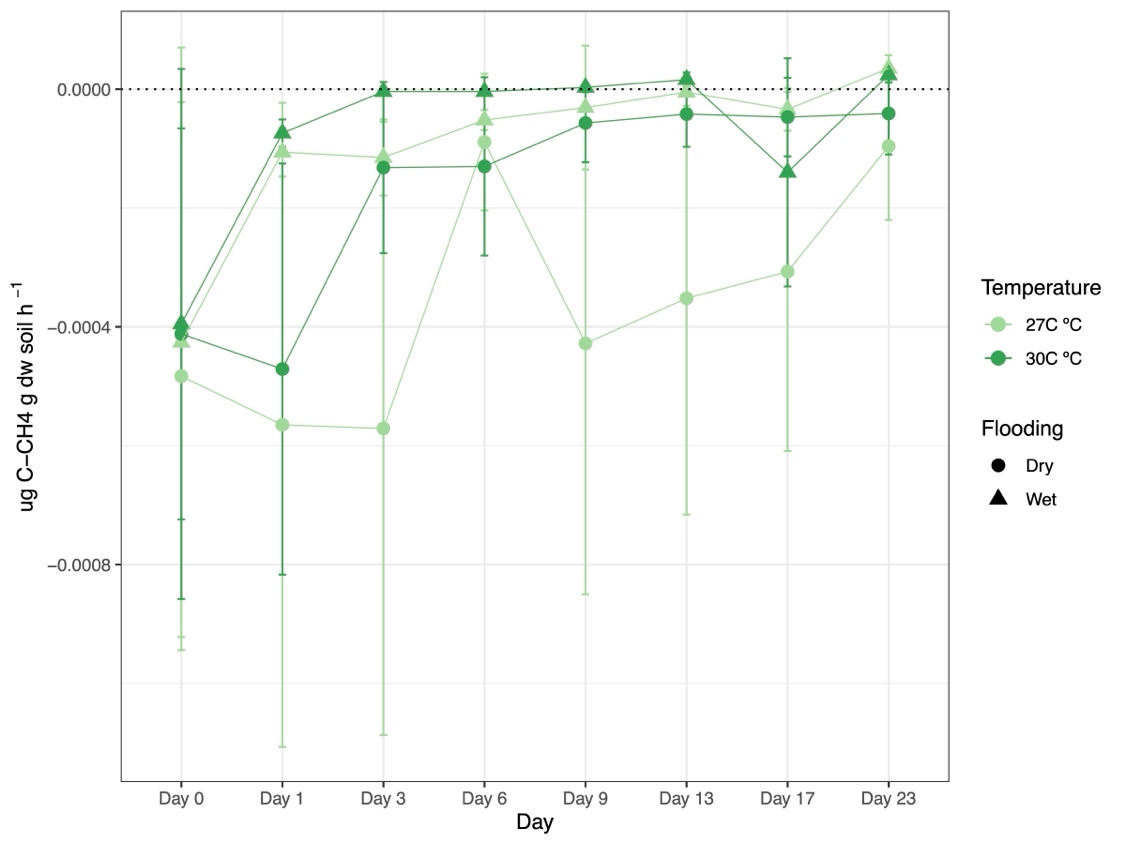


**Figure S5.** Detailed CH_4_ emission dynamics for PFO soils from Days 0 to 23. The soil samples were submitted to changes in flooding (wet and dry) and temperature (27 ºC and 30 ºC) conditions.


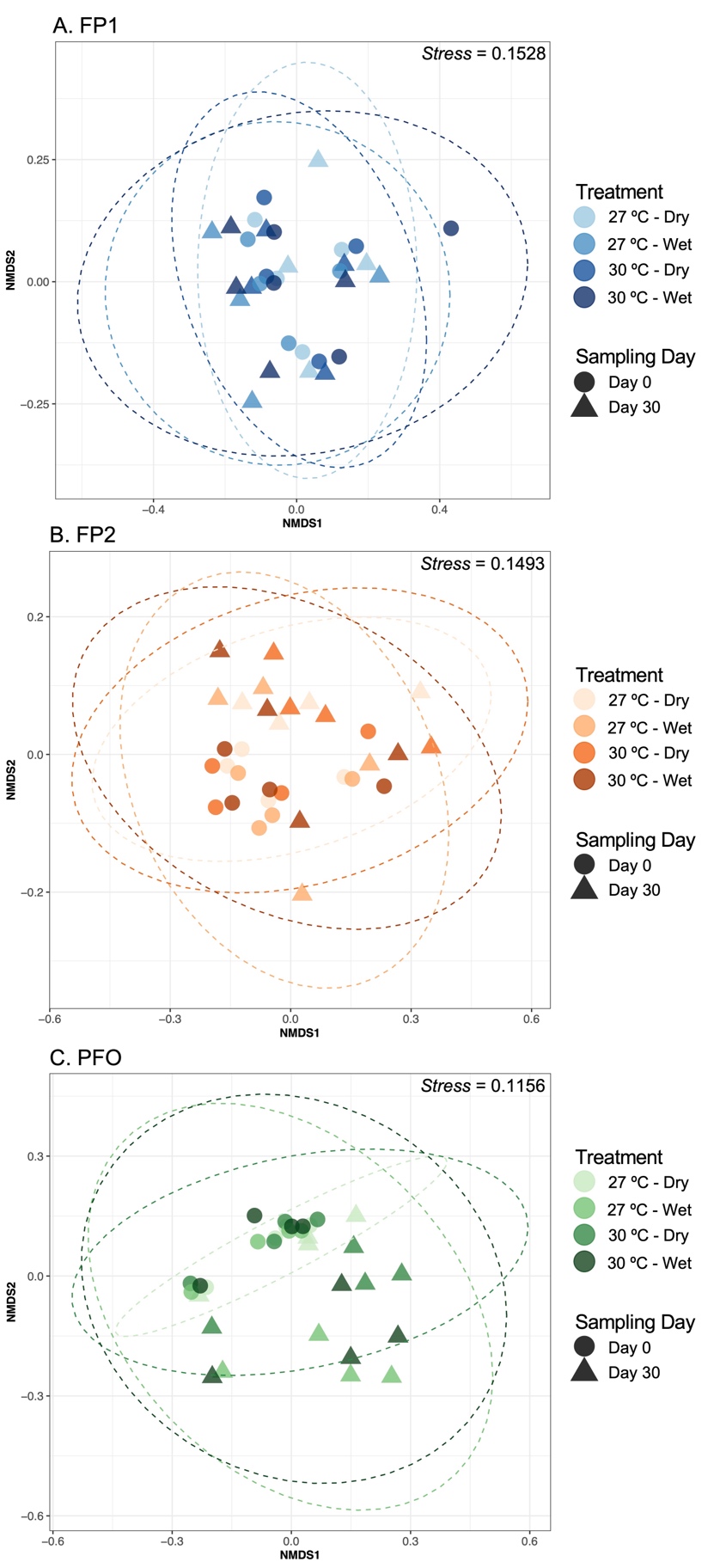


**Figure S6.** Clustering of the taxonomic structure of the floodplain 1 (A), floodplain 2 (B) and upland forest (C) soils under different conditions of flooding (wet and dry) and temperature (27 ºC and 30 ºC). Plot is based on the non-metric multidimensional scaling (NMDS) using the Bray-Curtis distance index.


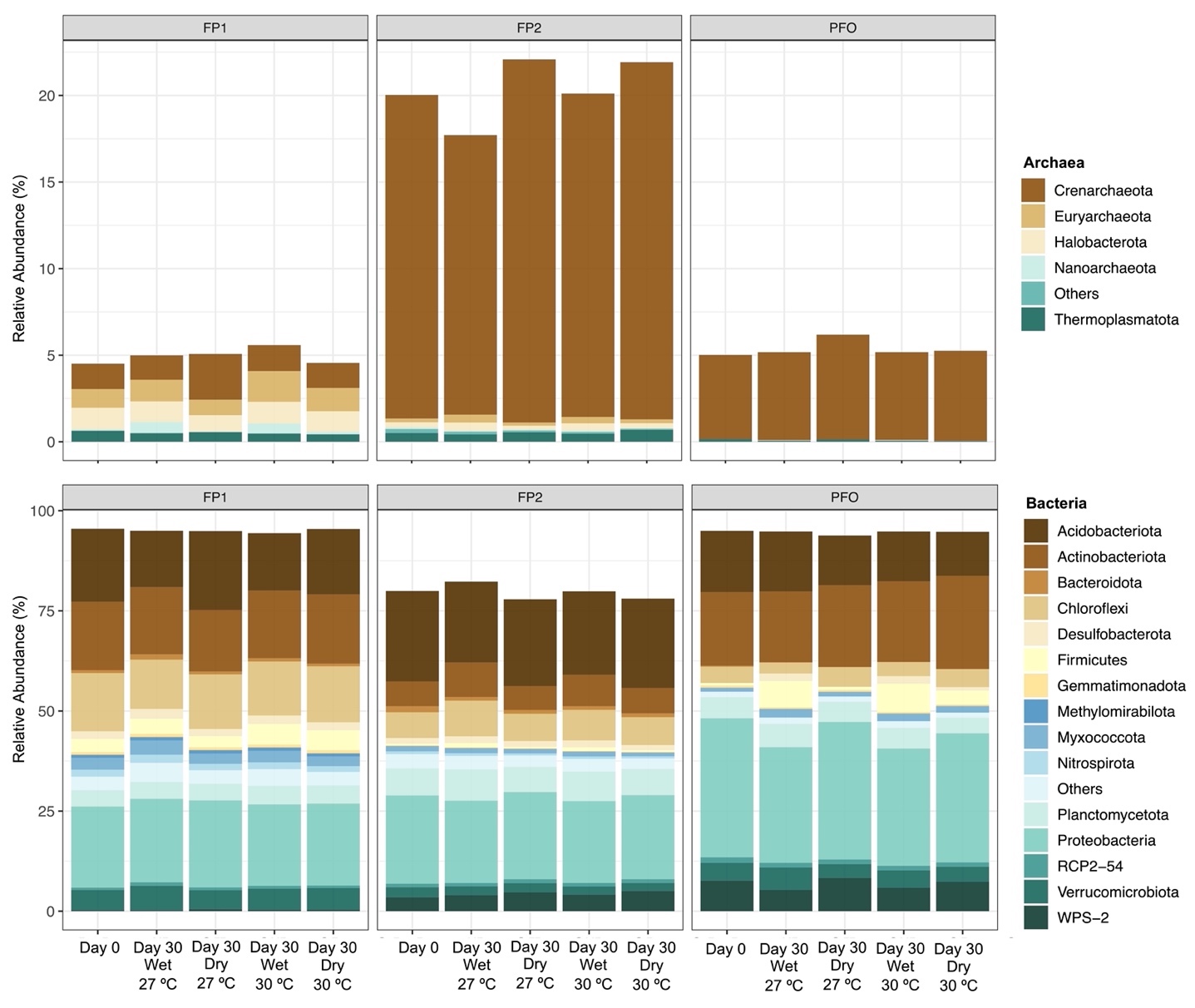


**Figure S7.** Relative abundance (means per treatment) of the Archaeal and Bacterial microbial communities in the floodplain (FP1 and FP2) and upland forest soils (PFO) under different conditions of flooding (wet and dry) and temperature (27 ºC and 30 ºC). Days 0 and 30 represents the first and last days of the experiment that the microbial communities where accessed.
